# I. Embryonal vasculature formation recapitulated in transgenic mammary tumor spheroids implanted pseudo-orthotopicly into mouse dorsal skin fold: the organoblasts concept

**DOI:** 10.12688/f1000research.2-8.v2

**Published:** 2013-07-11

**Authors:** Halina Witkiewicz, Phil Oh, Jan E Schnitzer

**Affiliations:** 1 Proteogenomics Research Institute for Systems Medicine, San Diego, CA, 92121, USA

## Abstract

Inadequate understanding of cancer biology is a problem. This work focused on cellular mechanisms of tumor vascularization. According to earlier studies, the tumor vasculature derives from host endothelial cells (angiogenesis) or their precursors of bone marrow origin circulating in the blood (neo-vasculogenesis) unlike in embryos. In this study, we observed the neo-vasculature form in multiple ways from local precursor cells. Recapitulation of primitive as well as advanced embryonal stages of vasculature formation followed co-implantation of avascular ( *in vitro* cultured) N202 breast tumor spheroids and homologous tissue grafts into mouse dorsal skin chambers. Ultrastructural and immunocytochemical analysis of tissue sections exposed the interactions between the tumor and the graft tissue stem cells. It revealed details of vasculature morphogenesis not seen before in either tumors or embryos. A gradual increase in complexity of the vascular morphogenesis at the tumor site reflected a range of steps in ontogenic evolution of the differentiating cells. Malignant- and surgical injury repair-related tissue growth prompted local cells to initiate extramedullar erythropoiesis and vascular patterning. The new findings included: interdependence between the extramedullar hematopoiesis and assembly of new vessels (both from the locally differentiating precursors); nucleo-cytoplasmic conversion (karyolysis) as the mechanism of erythroblast enucleation; the role of megakaryocytes and platelets in vascular pattern formation before emergence of endothelial cells; lineage relationships between hematopoietic and endothelial cells; the role of extracellular calmyrin in tissue morphogenesis; and calmyrite, a new ultrastructural entity associated with anaerobic energy metabolism. The central role of the extramedullar erythropoiesis in the formation of new vasculature (blood and vessels) emerged here as part of the tissue building process including the lymphatic system and nerves, and suggests a cellular mechanism for instigating variable properties of endothelial surfaces in different organs. Those findings are consistent with the organoblasts concept, previously discussed in a study on childhood tumors, and have implications for tissue definition.

***“The significant problems we face cannot be solved at the same level of thinking we were at when we created them”.***

*– Albert Einstein*

## Introduction

### Some of the unsolved problems in vascular biology

Tumor vascular biology research has been beleaguered with multiple controversial experimental data and unanswered questions despite the technological progress and new concepts in network biology. The importance of understanding interactions among individual elements of a system has only recently gained popularity. Much of the information on mapping of mammalian interactome networks waits to be experimentally validated, although advancements have been made in small model organisms (yeast, *Saccharomyces cerevisiae*; fruit fly, *Drosphila melanogaster*; and worm, *Caenorhabditis elegans*) ^1^. Richness and redundancy enabling the natural selection of evolving biological systems make their analysis difficult and call for new methods and ways of thinking ^2, 3^. Unsolved problems include lineage relationship between endothelial stem cells (SCs) and hematopoietic stem cells (HSCs) ^4, 5^; the role of platelets in embryonal development ^6, 7^; lineages of immune cells ^8^, tolerance of tumors by the host immune system ^9^ and reprogramming of energy metabolism by tumors ^5, 9^. The question of biological significance of the cellular heterogeneity within tumors also remains unanswered.

### Vasculogenesis versus angiogenesis

Traditionally vasculogenesis and hematopoiesis were subjects of independent studies and some fundamental issues have remained unclear in each case. The origin of the precursors of endothelial cells (ECs) has created controversy in understanding tumor growth related vasculogenesis, whereas the illusive origin of HSCs had been a daunting problem in understanding embryonal development. As opposed to *de novo* vasculature formation during embryogenesis (vasculogenesis), tumor vasculature has been thought to emerge by expansion of the host vasculature through postnatal sprouting of endothelial cells (angiogenesis). Therefore, it was proposed that inhibiting angiogenesis would stop tumor growth ^10^. When that conceptually simple objective turned out to be impossible to reach, a notion of neo-vasculogenesis was born, suggesting that tumor vessels form from circulating bone marrow-derived endothelial precursors rather than from mature ECs ^11^. However, the views on tumor neo-angiogenesis remained polarized ^3, 12^.

### Erythropoiesis

Descriptions of the two main types of erythropoiesis, i.e. primitive and definitive, has kept changing over the years. Extremely large nucleated cells and smaller enucleated ones, distinguished first, were later referred to as nucleated and anuclear, respectively ^13, 14^. Each wave of erythropoiesis was reported to generate more potent progenitors (progenitors of more numerous cell types) ^15^. Lately, five distinct classes of hematopoietic cells in murine conceptus emerged from studies on their activity in transplantation or clonogenic assays *in vitro*: primitive; pro-definitive (myeloid progenitors); meso-definitive (lymphoid-myeloid progenitors); meta-definitive (neonatal repopulating HSCs); and adult-definitive (adult repopulating HSCs). Maturation of primitive, nucleated precursors from yolk sac in circulation has also been reported ^16^. These classes of cells appeared to be generated independently of each other in distinct hematopoietic sites ^17^. In other words, the origins of HSCs seemed to be multiple. Conclusions on the embryonic location of HSCs capable of long-term repopulation depended on the assays used to test them. Those from yolk sac repopulated neonate recipients better than adults, whereas those from aorta-gonad-mesonephros (AGM) repopulated adult recipients better than neonates ^18^. Evidently, the successful repopulation requires a certain level of developmental compatibility. After birth, bone marrow ( *medulla ossea*) was proposed to be the proper niche where the formation of blood elements would continue for the lifetime as needed. Therefore, extramedullar hematopoiesis observed in liver ^19, 20^, adipose tissue ^21^, cerebellar hemagioblastoma ^22^, meningioma ^23^, or subdural hematoma ^24^ was perceived as unusual. However, in our study, it appeared essential for tumor growth and for regeneration of normal tissues.

### Erythrocytic enucleation

The debate over the mechanism leading to the elimination of the nucleus from maturing erythroblast during erythropoiesis, expulsion of nucleus or karyolysis, dated back to the late nineteenth century ^25^. The current consensus on achieving enucleation by nuclear extrusion came from the ultrastructural studies published in 1967 that addressed the controversy by looking at either peripheral blood from dogs intentionally made anemic ^26^ or at erythroid clones grown in the spleen of irradiated mice transplanted with syngeneic marrow cells ^25^. In normal peripheral blood or non-manipulated spleen, the enucleation event was too rare to study by transmission electron microscopy (TEM). Ultrastructural analysis of a developing embryo are not available in the literature, even for an organism as well studied as the mouse. The Edinburgh Mouse Atlas Project offers no TEM images ( http://www.emouseatlas.org/emap/home.html). In our model, the enucleation event was frequent enough to reveal its mechanism and we found no evidence for nuclear extrusion. Instead, we observed several variants of generating erythrocytes by nucleo-cytoplasmic conversion. Thus, depending on the model, the same method could generate conflicting results. Interestingly, the analysis of chromosomal topology showed that at certain stages of erythroblast differentiation, two chromosomes were unidentifiable ^27^. Below, we use the term "erythrosome" as a synonym for "erythrocyte" because the latter is not a cell.

### Parallels between tumor and embryo

Similarities and differences in the antigenic composition of tumors and embryonal tissues, as well as in patterns of isozymes distribution, have been studied for years ^28, 29^. Reappearance in tumors of embryonic and fetal antigens, as well as isozymes, has been shown to be a common phenomenon, yet similar alterations have sometimes been observed in non-neoplastic tissues under dietary and hormonal influences ^29, 30^. In cases of carcinoembryonic antigen (CEA), the concept ultimately did not apply, contradicting the antigen’s name, as CEA-cross-reactive antigens were located in normal human tissues including blood ^31^. Also, "patterned vascular channels without EC", i.e. vascular mimicry, were observed in embryos as well as in growing tumors ^32, 33^. The analogy between the two types of growing tissues was further enforced by showing that tumor cells could be epigenetically reprogrammed into normal cell types ^34, 35^. Here, we present an unprecedented collection of original images documenting the full range of growing dynamic complexity of the tumor-induced vasculature formation, reminiscent of the embryonal one.

### Definitions of stem cells

The hallmark of the classic definition of adult TSCs is the potential of the cells to self-renew and differentiate ^36^. The 2002 amendment to that definition ^37^ emphasized additional aspects of the concept, namely: a shift from the cellular view to a system view including self-organizing processes, stemness as a capability rather than as a cellular property, dependence on the growth environment, within-tissue plasticity, and the functionality of the TSCs and the tissue. The stemness as an attribute of a system, rather than of a particular cell lineage, questioned the usefulness of presumed protein markers for identifying stem cells (SCs) ^37– 39^. With regard to both embryonal and cancer SCs (ESCs and CSCs, respectively) the views remain opposing. On the one hand, neither ESCs nor CSCs fulfill all those criteria ^40^; on the other hand, both do but with certain qualifications. In the case of the ESCs, self-renewal and differentiation is likely to occur sequentially for two reasons. (1) The redundancy during development could serve as an evolutionary safety measure; in the context of the early embryo, that could mean generating a certain number of identical cells before their differentiation begins ^41^. (2) Heterogeneity with respect to cell cycle activity was detected even in such an iconic cell lineage as the HSCs of adults ^42^. In case of the CSCs, their role in tumorigenesis appeared to depend on microenvironment as discussed below and in one of our accompanying articles ^43^.

### The goal and significance of this study

The goal of our study was to visualize the cellular mechanisms of vasculature formation associated with the growth of malignant tissue. We carried out the ultrastructural *in situ* analysis of initially avascular breast tumor spheroids co-implanted with homologous tissue graft, into the mouse dorsal skin folds ^44, 45^. A complementary study on such spheroids implanted without the graft is the subject of a separate report ^43^. In contrast to the reductionism that dominated the ultrastructural studies of the second half of the last century, we analyzed tissue sections instead of isolated tissue components. The high resolution was necessary to investigate distinct physiology and functions of individual cells whereas the tissue context unveiled interactions among the cells. The results revealed interdependence between erythrogenesis and vasculogenesis localized at the site of tissue growth or repair and other new qualities of the emerging system. The data illustrated a great variability and increasing complexity of the vasculature morphogenesis, i.e., formation of blood and vessels, as both were evolving from primitive forms while enabling the tissue building process. Like in the embryo ^17^, the new tumor vasculature was developing independently of the existing one. New erythrosomes and other blood elements formed extramedullary, and new vessels were assembling around them before initiation of the blood flow occurred. Vessel "sprouts" progressed toward other vessels not away from them. By suggesting local tissue cells as progenitors of the new vasculature, those observations related to the concept of tissue stem cells (TSCs).

The results of the morphological analysis of the vasculature formation in our model were consistent with the amended functional definition of the TSCs concept. They are not in harmony with the common understanding of the cell lineages as hierarchical and irreversible progress of differentiation. Moreover, the new findings suggest a hypothesis that the adult vasculature formation represents a partial recapitulation of the embryonal organogenesis involving coordinated activity of some cells capable to form all tissue types of a particular organ *de novo*. Such a perspective is not entirely new. The term "organoblast" was used in 1997 in reference to pediatric salivary gland tumors that had morphological characteristics of the organ with maturation arrested at a primitive state of development ^46^. Later, mouse mammary SCs in the form of *in vitro* grown mammospheres ("organoids") were shown to regenerate morphologically normal mammary epithelial ducts after implantation into mammary fat pads surgically cleared of the endogenous epithelium ^47^.

Our results also demonstrate the potential of immunocytochemistry to reveal further molecular-level details of the cellular interactions as they relate to regional tissue growth. This approach is suitable to validate concepts derived from proteogenomics as well as effects of pharmacological treatments. It is far more relevant to medical problems than *in vitro* studies and cheaper than conducting clinical trials.

## Materials and methods

The study was performed according to protocols approved by Sidney Kimmel Cancer Center’s (SKCC) OLAW-approved Institutional Animal Care and Use Committee (Assurance No A4128-01). The protocol numbers were: 03–16A and 05–11 for Grants CA104898 and CA119378, respectively. No human specimens were involved in any of the experiments outlined here.

### Acronyms

AGM: aorta-gonad-mesonephros

ATP: adenosine tri-phosphate

BMP4: bone morphogenetic protein 4

CEA: carcinoembryonic antigen

CIB1: calcium & integrin binding protein 1

CSCs: cancer stem cells

ECM: extracellular matrix

ECs: endothelial cells

ER: endoplasmic reticulum

ESCs: embryonal stem cells

GFP: green fluorescent protein

H2B: histone 2B

HSCs: hematopoietic stem cells

OLAW: Office of Laboratory Animal Welfare

RT: room temperature

SCs: stem cells

TEM: transmission electron microscopy

TIMPs: with tissue inhibitors of metalloproteinases

TSCs: adult tissue stem cells

VVOs: vesiculo-vacuolar organelles

### Animals

Host and graft donor, female, athymic nude mice, 8–9 weeks old, were purchased from Harlan. The donor mouse with GFP-labeled ubiquitin was from The Jackson Laboratory (Stock Number: 004353; Strain Name: C _57_BL/6-Tg(UBC-GFP) _30_Scha/J). The mice were housed in the SKCC animal care facility The mice were housed in the SKCC animal care facility with controlled 12/12 hr light/dark cycle and temperature maintained at + 22°C. The mice were on Tekand global 14% protein rodent diet (Harlan) with access to water ad libitum. For surgery, they were anesthetized (7.3 mg ketamine hydrochloride and 2.3 mg xylazine/100 g body weight, inoculated i.p.) and placed on a heating pad. Immediately before tissue harvesting the tumor hosting mice as well as the graft donors were euthanized with pentobarbital overdose (100 mg/kg i.p.).

### Cell lines

The parental murine breast cancer cell line, N202.1A ^48^ was stably transfected to express GFP under histone H2B promoter ^49^. The two cell lines, N202.1A parental and N202.1A+H2B-GFP (obtained from Drs. J. Lustgarten and P. Borgstrom) were used to form tumor spheroids by culturing 2×10 ^5^ cells per well for 2–3 days prior to implantation.

### Chambers

A week after establishment of mouse dorsal skin chambers, the tumor spheroids were implanted on a pad of homologous tissue, namely, minced breast fat pad from a lactating mouse (pseudo-orthotopically) as described earlier ^45^. The tumors were incubated in the chambers for one or three weeks ( Table 1). Their final size was about 1–3 mm in diameter. The blood circulation in tumor vessels was monitored *in vivo* by light microscopy before disassembling the chambers ^50^.

**Table 1.** Experimental design.

Figure numbers	Host mouse no	Tumor cell line	Graft tissue	Incubation time (days)
1, 4 (A-D & F), 5 (A, B, E, F), 6, 7 & 15 (A, B)	5 (hyaline membrane)	N202.1A parental	Ubiquitin-GFP breast fat pad	5
2, 3, 4 (E & G-I), 5 (C, D) & 8 (A-H), 15 (C-F)	5	N202.1A parental	Ubiquitin-GFP breast fat pad	5
9, 10 (C), 15 (C)	3	N202.1A+H2B-GFP	Breast fat pad	22
10 (A, B), 11–14, 15 (D, E, F)	2	N202.1A+H2B-GFP	Breast fat pad	21

### Antibodies

The GFP-specific rabbit polyclonal IgG (ab290) was from Abcam; CIB1-specific rabbit polyclonal IgG (11823-1-AP) was from ProteinTech Group, Inc.; CD34-specific rat monoclonal IgG _2a_ (sc-18917) and non-reactive goat polyclonal IgG (sc-34284) were from Santa Cruz.

### Tissue processing

The tumors with some surrounding tissues were dissected out and cut in halves perpendicularly to the host skin surface while immersed in cold fixative (4% paraformaldehyde in 0.1 M Na cacodylate pH 7.4). The skin region served later as a reference to distinguish between edges of the tumor facing the skin and those facing the glass window of the chamber. The halves were then separated and processed independently for TEM and immunocytochemistry.

### TEM

The tissues were transferred into a stronger fixative (4% paraformaldehyde/2.0% glutaraldehyde in 0.1 M Na cacodylate pH 7.4) to better preserve the ultrastructures before further cutting. They were cut into 1 mm thick slices in planes perpendicular to the plane of the first cut and to the skin surface, finally, into ~ 1 mm ^3^ blocks, transferred into a fresh portion of the fixative in which they were cut and incubated for 2 hrs at 4°C. The fixed tissue blocks were washed with 0.1 M Na cacodylate – HCl buffer pH 7.4 (3 × 15 min.) and post fixed in 1% OsO _4_ in 0.1 M Na cacodylate buffer, pH 7.0 for 60 min. on ice, washed with water and stained with 1% uranyl acetate at room temperature (RT) for one hour. The blocks were embedded in EMbed-12 (EM Sciences, Cat. No 14120). The resin embedded tissues were cut into 60 nm sections, using a Leica Ultracut UCT ultramicrotome, and stained with lead citrate ^51^ or viewed without further contrasting.

### Immunocytochemistry

During cutting into ~1 mm ^3^ blocks as described above, the tissues were kept in the mild fixative to protect antigenic epitops (4% paraformaldehyde in 0.1 M Na cacodylate pH 7.4). The tissue blocks were vitrified by infiltrating the pieces with 50% PVP (polyvinylpyrrolidone) containing 2.3 M sucrose in 0.1 M Na-cacodylate buffer, pH 7.4, for 2 hrs or over night, mounted on metal pins and frozen in liquid nitrogen, as described by Tokuyasu ^52^. Frozen tissues were cut into 70 nm sections, using a Leica Ultracut UCT ultramicrotome with the cryo-attachment. The sections were picked from the knife with 2.3 M sucrose and floated on 1% ovalbumin (Sigma, Cat No.A5378) in 0.1 M Na-cacodylate buffer for at least one hour before incubation with specific or non-reactive antibody (50 µg/ml) at RT for one hour. Sections were then rinsed eight times with 0.1% ovalbumin in the same buffer and incubated for one hour with 10 nm Au coupled to protein A (from Dr G. Posthuma; Cell Microscopy Center, university Medical Center Utrecht, The Netherlands). The eight rinsing steps were repeated before fixation of the immune complexes with 1% glutaraldehyde. After rinsing three times with water, the immunostained cryosections were contrasted with a mixture of uranyl acetate and methyl cellulose (25 centipoises, Sigma M-6385) in water, at a final concentration of 1.3% each, for 10 min. Excess liquid was removed and the sections were dried at RT.

### Viewing

All sections were viewed and the images captured at 100 kV using a Morgagni 268 electron microscope equipped with MegaView III digital camera. Analyzed tissue sections were first examined at low magnification and coordinates for each hexagonal sector of a grid covered with tissue were recorded automatically. Subsequently all sectors were explored at least once at variable high magnifications. Interesting images were captured at the magnification best suited to document a particular phenomenon or structure, including colloidal Au grains. The images were transmitted from the microscope camera to an iTEM imaging platform from Olympus Soft Imaging Solutions and archived in a designated database. In some cases, the final images were assembled by multiple image alignment (MIA) to increase the surface area without losing the resolution. We used the graphics editing program, Adobe PhotoShop, to add cell type-specific color-coding shown in the twin set of images included in the Supplement.

## Experimental design

Three of the five recipient mice used in this and two accompanying articles were assessed here, as summarized in Table 1. The same numbering system was used in all three articles. The other two mice had the tumor spheroids implanted without the graft. The same numbering system was used in all three articles. Three weeks after implantation, we observed a range of stages of vasculature formation simultaneously due to continuous growth of the tumors. Cells responding to locally variable microenvironment were not synchronized. We added the five days incubation time to see if other time points were necessary to extend that range. Indeed, different yet overlapping sets of options were found. However, between the two types of implantation, with & without the graft, the range of options for tumor supporting vasculature formation was well represented three weeks after implantation. Therefore, we concluded that additional time points were not necessary for our purpose. Yet, it was at the short incubation time that the hyaline membrane between the tumor & the glass remained unattached to the underlying tissues. That fragile membrane collapsed into a viscous droplet on the glass wall of the chamber at the time of tissue harvest and was analyzed because it appeared to contain a red, vascular pattern-resembling network when viewed through dissecting microscope lenses. Images from the hyaline membrane introduced additional observations regarding morphogenesis of the fibrous tissue on top of the implanted tumor, including vasculature formation in the non-malignant tissue.

## Results

Multiple steps of cellular differentiation and the growing complexity of the vasculature formation process were observed in tissue samples harvested at only two different time points (5 and 21 days after implantation) due to local variability resulting from lack of synchronization *in vivo*. The emergence of increasingly more specialized phenotypes of individual cells characterized those steps, signifying progress in the cellular differentiation. Multiple forms of erythropoiesis reflected the multiple levels of cell maturation.

**Figure 1.**
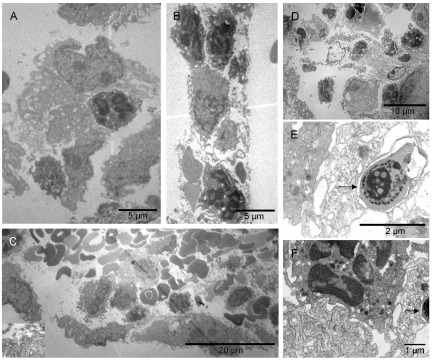
A blueprint for vascular pattern created by migrating erythroblasts and megakaryocytes. The pattern-forming heterogeneous population of the cells migrating in the hyaline membrane consisted of (a) those with large nuclei occasionally containing double nucleoli and with asymmetrically distributed, irregularly shaped, large cytoplasm and satellite platelets i.e. megakaryocytes, and (b) those with atypical, lobular, heterogeneously dark nuclei and dark cytoplasmic granules in the absence of mitochondria, usually accompanied by erythrosomes, i.e. most likely erythroblasts [ **A**]. The four elements, cellular and sub-cellular (erythrosomes and platelets), appeared evenly dispersed among each other like in the narrow (about capillary short dimension) column in [ **B**] or the megakaryocytes that displayed a tendency to migrate to the periphery of the larger cluster and surround the other elements [ **C**]. Those poorly differentiated cells were probably bipotent, megakaryocyte and erythroid, progenitors [ **C**]; compare with Figure 8 in ^55^. The cellular and sub-cellular elements appeared to be held together with extracellular fibrous components, possibly proteoglycans and little collagen (the insert in [ **C**]). The resulting red pattern appeared as a vascular network under low magnification of dissecting microscope. The parallel arrangement of extracellular, short, non-collagen fibers suggested mobility for the cells secreting them [ **D**]. The extra-cellular matrix (ECM) producer [ **E**] located to the right of the erythroblast [ **F**] contained a composite dense body (arrows in [ **E** & **F**]) characteristic of platelets (Figure 111-1, E in ^56^).

**Figure 2.**
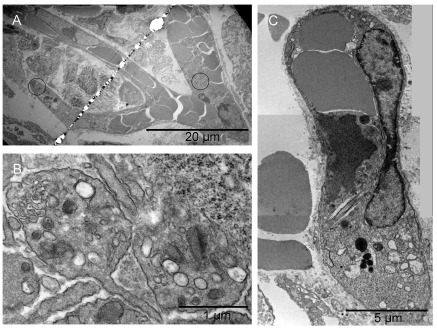
Primitive vascular pattern formed by megakaryocytes without endothelium. Primitive vascular pattern in the tumor-harboring solid tissue below the hyaline membrane consisted of megakaryocytes and platelets engaged in direct and tight contact with one another and with erythrosomes and erythroblasts. There were no ECM-filled spaces left between them nor was there an additional cellular layer covering those elements. The elongated branching structure in [ **A**] appeared kept together by megakaryocytes and platelets (N.B. the line in the middle of the image is a scratch caused by an imperfection of the knife-edge during cutting the section). In the two circled regions of that structure, incomplete separation of platelets from megakaryocytes provided ultrastructural evidence for their identity and for an ongoing process of generating platelets at that location. One region was the bifurcation area at the base of the right almost vertical branch, enlarged in [ **B**] and the other was the tip of the lower left branch (enlargement not shown). Evidently, megakaryocytes were capable of organizing erythrosomes into branching primitive vascular structure before ECs emerged to provide protective walls of a vessel. In a smaller elongated structure of similar nature, although without platelets in the section plane, one of the cells contained an Auer body [ **C**], previously described in neutrophils from cases of leukemia and defined as azurophilic, large, elongated granules (Figure 64-10 in ^57^). The upper part of that cell contained two erythrosomes; whereas what remained of the nucleus had a different texture compared with that of the nucleus in the second cell of that complex. The prominent dark granules of the latter resembled those in the cell next to the megakaryocyte from the tip of the lower branching structure in [ **A**]. If the irregularly shaped seemingly empty space next to those granules was a prototype of a lumen then both those cells could have potentially been angioblasts.

**Figure 3.**
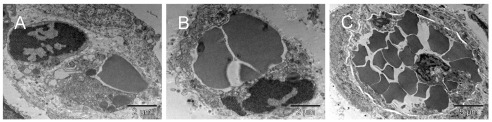
Extramedullar erythropoiesis via nucleo-cytoplasmic conversion. The single cells undergoing structural remodeling in the nucleus and cytoplasm [ **A** & **B**] represented at slightly different stages of erythropoiesis. Conceivably, a few such cells, when gathered into a cluster, could fill up a larger space with erythrosomes creating a cobble stone pattern, also called ‘erythropoietic lake’ or myeloplaxes [ **C**]. Platelets and megakaryocyte were recognizable at the periphery of the cluster.

**Figure S1.**
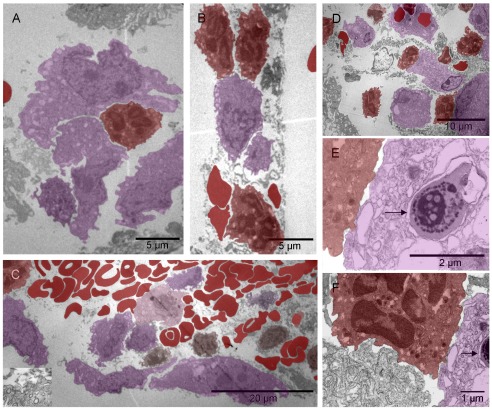
A blueprint for vascular pattern created by migrating erythroblasts and megakaryocytes. The pattern-forming heterogeneous population of the cells migrating in the hyaline membrane consisted of (a) those with large nuclei occasionally containing double nucleoli and with asymmetrically distributed, irregularly shaped, large cytoplasm and satellite platelets i.e., megakaryocytes (purple), and (b) those with atypical, lobular, heterogeneously dark nuclei and dark cytoplasmic granules in the absence of mitochondria, usually accompanied by erythrosomes (red), i.e. most likely erythroblasts (light red) [ **A**]. The four elements, cellular and sub-cellular (erythrosomes and platelets), appeared evenly dispersed among each other like in the narrow (about capillary short dimension) column in [ **B**] or the megakaryocytes that displayed a tendency to migrate to the periphery of the larger cluster and surround the other elements [ **C**]. Those poorly differentiated cells were probably bipotent, megakaryocyte and erythroid, progenitors (brown) [ **C**]; compare with Figure 8 in ^55^. The cellular and sub-cellular elements appeared to be held together with extracellular fibrous components, possibly proteoglycans and little collagen (the insert in [ **C**]). The resulting red pattern appeared as a vascular network under low magnification of dissecting microscope. The parallel arrangement of extracellular, short, non-collagen fibers suggested mobility for the cells secreting them [ **D**]. The extra-cellular matrix (ECM) producer [ **E**] located to the right of the erythroblast [ **F**] contained a composite dense body (arrows in [ **E** & **F**]) characteristic of platelets (see Figure 111-1, E in ^56^).

**Figure S2.**
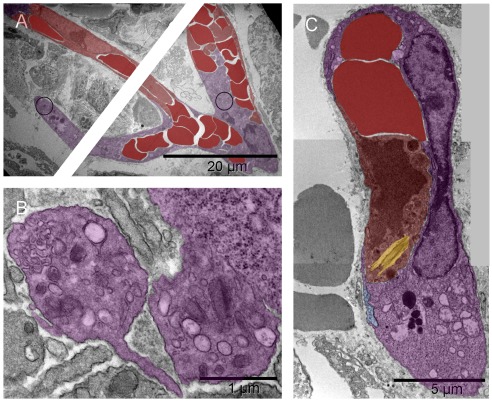
Primitive vascular pattern formed by megakaryocytes without endothelium. Primitive vascular pattern in the tumor-harboring solid tissue below the hyaline membrane consisted of megakaryocytes and platelets (purple) engaged in direct and tight contact with one another and with erythrosomes and erythroblasts (red). There were no ECM-filled spaces left between them nor was there an additional cellular layer covering those elements. The elongated branching structure in [ **A**] appeared kept together by megakaryocytes and platelets (N.B. the cutting imperfection mentioned in Figure 2 has been removed here). In the two circled regions of that structure, incomplete separation of platelets from megakaryocytes provided ultrastructural evidence for their identity and for an ongoing process of generating platelets at that location. One region was the bifurcation area at the base of the right almost vertical branch, enlarged in [ **B**] and the other was the tip of the lower left branch (enlargement not shown). Evidently, megakaryocytes were capable of organizing erythrosomes into branching primitive vascular structure before ECs emerged to provide protective walls of a vessel. In a smaller elongated structure of similar nature, although without platelets in the section plane, one of the cells contained an Auer body [ **C**], previously described in neutrophils from cases of leukemia and defined as azurophilic, large, elongated granules (Figure 64-10 in ^57^). The upper part of that cell contained two erythrosomes; whereas what remained of the nucleus had a different texture compared with that of the nucleus in the second cell of that complex. The prominent dark granules of the latter resembled those in the cell next to the megakaryocyte from the tip of the lower branching structure in [ **A**]. If the irregularly shaped seemingly empty space next to those granules was a prototype of a lumen then both those cells could have potentially been angioblasts.

**Figure S3.**
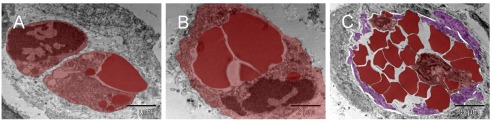
Extramedullar erythropoiesis via nucleo-cytoplasmic conversion. The single cells undergoing structural remodeling in the nucleus and cytoplasm represented at slightly different stages of erythropoiesis [ **A** & **B**]. Conceivably, a few such cells (red), when gathered into a cluster, could fill up a larger space with erythrosomes creating a cobble stone pattern, also called ‘erythropoietic lake’ or myeloplaxes [ **C**]. Platelets and megakaryocyte (purple) were recognizable at the periphery of the cluster.

Five days after implantation, in hyaline membrane between the tumor surface and the glass chamber wall, the earliest event seen was migration of megakaryocytes and erythroblasts and their products, platelets and primitive erythrosomes respectively, along with the bipotent precursors of both cell types ^7, 53– 55^ ( Figure 1 & Figure S1). The cells seemed loosely bound together by fibrous (non-collagen) secretion, likely proteoglycans. They appeared to be migrating in columns and differentiating simultaneously, as if scouting the hypoxic avascular space under the glass and establishing a blueprint for vascular pattern. A composite dense body resembling those seen in platelets ^56^ was found in one of the cells depositing elements of ECM ( Figure 1 [E & F]) and suggested that the cell building ECM was related to the cell type producing platelets, i.e., to megakaryocyte. At the same time on the tumor surface, the two cell types had formed a primitive vascular pattern in the absence of ECs ( Figure 2 & Figure S2). A crystalline inclusion resembling an Auer body ^57^ was found in a small structure of such nature ( Figure 2 [C]). The formation of vasculature attached to the tumor was similar to that of tumors incubated for three weeks without the graft (Figure 1 [D] in the accompanying article II). That similarity represented an overlap in terms of the earliest stages of vasculature morphogenesis found at incubation times two weeks apart. In both cases, the cells represented two principal functional attributes of the vasculature. The first attribute, the metabolism-related functions of transporting gases (O _2_ & CO _2_) and of generating ATP anaerobicly ^58^, were associated with erythrosomes. The second one, the structure-organizing functions, were represented initially by pro-megakaryocytes in the form of secreted elements of the extracellular matrix (ECM), possibly carcinoembryonic antigen (CEA) ^31^ and later, by mature megakaryocytes and platelets. That way, megakaryocytes were in a position to receive additional nourishment (ATP) from the erythrosomes as their mitochondria suffered from some hypoxic distress ( Figure 1 & Figure S1 [F]). ECs were absent. By shaping the vascular pattern, megakaryocytes presented themselves as a cell type involved in early vasculature morphogenesis, in addition to the known later role of platelets in mending vascular injuries by their aggregation and the thrombus formation. At higher magnification, the emerging erythrosomes revealed nucleo-cytoplasmic conversion as the mechanism of enucleation in single erythroblasts as well as in clusters of them ( Figure 3 & Figure S3).

**Figure 4.**
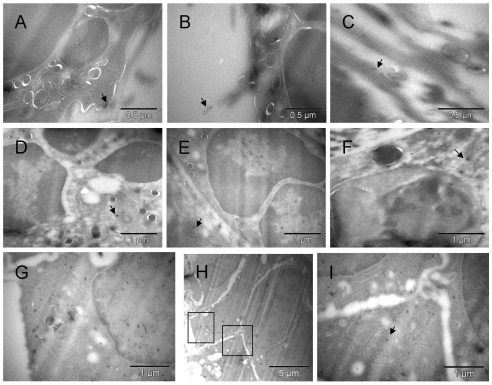
HSCs and their progenitors at the tumor site labeled with CD34 marker. Clusters of the gold label were found on the cell surface, in the nucleus, cytoplasm and mitochondria of cells morphologically fitting the description of erythroblasts [ **A**, **B**, **D** & **E**] and on extracellular collagen fibers produced by them [ **B** & **C**]. A fragment of cell with mixed phenotype is shown in [ **F**]. The cluster of CD34-labeled giant erythroblasts (potentially leading to myeloplaxes) contained one cell with a double nucleus (right side in [ **H**]). Two regions of [ **H**] are enlarged in [ **G** & **I**], left and right respectively, to show oversized mitochondria, some converting into peroxisomes [ **G**]. That cluster of cells was bigger than the one shown earlier ( Figure 3C), and the presence of mitochondria despite clear commitment to erythrogenesis suggested that here the process was more efficient. Arrows point out some clusters of Au grains.

**Figure S4.**
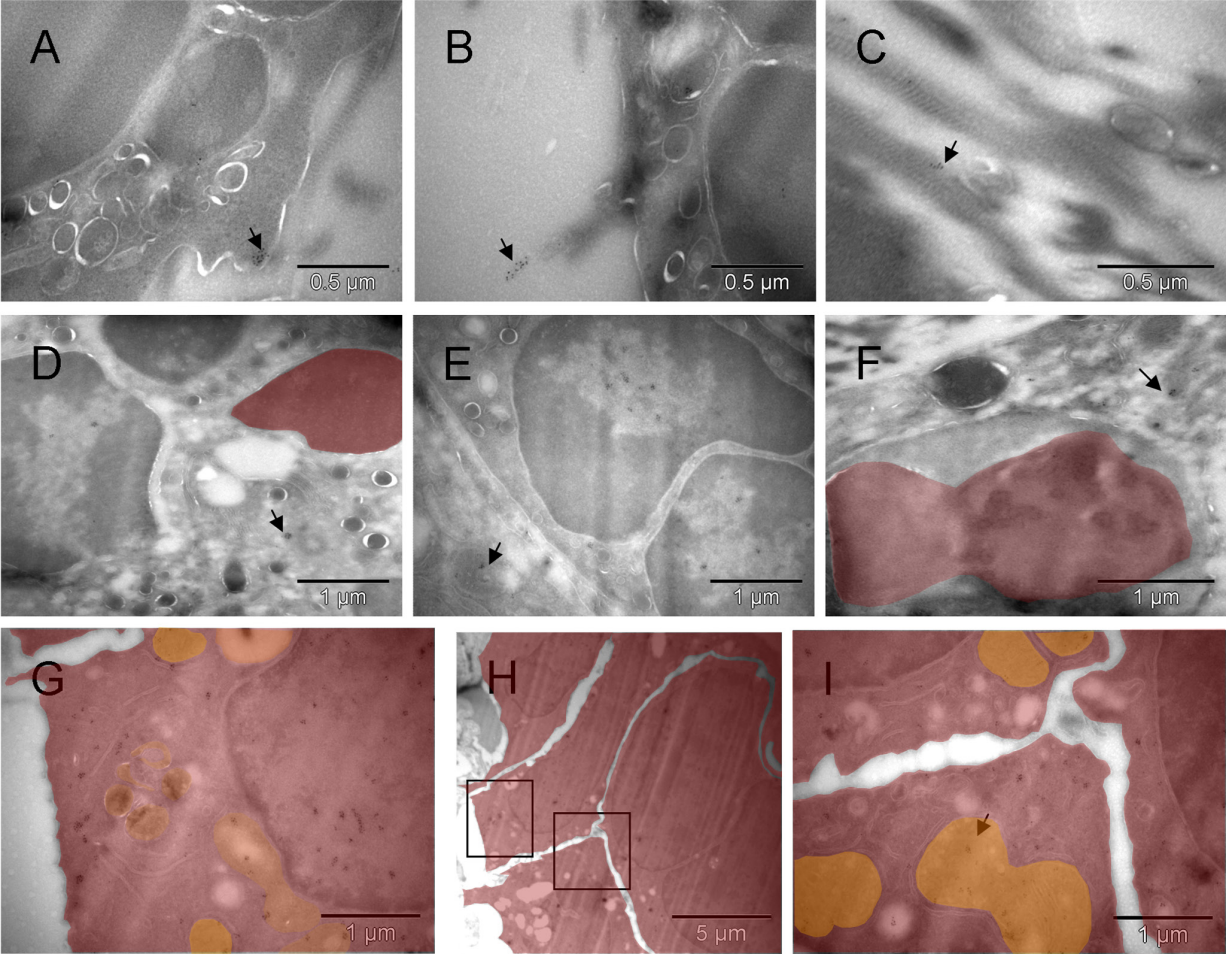
HSCs and their progenitors at the tumor site labeled with CD34 marker. Clusters of the gold label were found on the cell surface, in the nucleus, cytoplasm and mitochondria of cells morphologically fitting the description of erythroblasts [ **A**, **B**, **D** & **E**] and on extracellular collagen fibers produced by them [ **B** & **C**]. A fragment of cell with mixed phenotype is shown in [ **F**]. The cluster of CD34-labeled giant erythroblasts (red), potentially leading to myeloplaxes, contained one cell with a double nucleus (right side in [ **H**]). Two regions of [ **H**] are enlarged in [ **G** & **I**], left and right respectively, to show oversized mitochondria (orange), some converting into peroxisomes (dark & orange) [ **G**]. That cluster of cells was bigger than the one shown earlier ( Figure S3C), and the presence of mitochondria despite clear commitment to erythropoiesis suggested that here the process was more efficient. Arrows point out some clusters of Au grains.

The immunohistochemical staining detecting CD34 confirmed the erythropoietic lineage identity of the cells by analyzing frozen counterparts of the samples shown in Figure 1, Figure 2, Figure 3 & Figure S1, Figure S2, Figure S3 (Mouse 5, and some from Mouse 3). The CD34 label was present in mononuclear and giant polyploidal erythroblasts ( Figure 4 & Figure S4 [A-F] & [G-I] respectively). Both types were at the early stage of nucleo-cytoplasmic conversion but in the giant cells, the mitochondria were still intact and they were oversized. The few giant cells belonged to a larger cluster of such cells, most likely representing cells undergoing growth and endoreduplication before converting into giant erythroschizonts that would later break into erythroschizomers. Together those features suggested very efficient production of large number of erythrosomes. In 1909, Maximow interpreted the emergence of such extremely large primitive nucleated red cells found in mammalian embryos as a response to relative hypoxia of the fetus and the need for rapid generation of a large amount of blood ^13^. The extramedullar erythropoiesis induced by hypoxia of growing tumors resembled fetal erythropoiesis in that regard.

**Figure 5.**
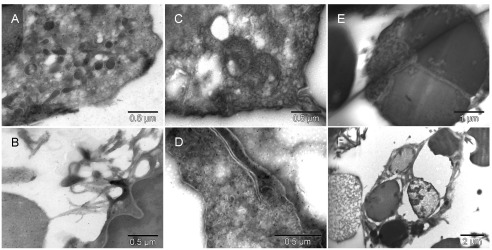
Cells of graft origin labeled with GFP under ubiquitin promoter. GFP-specific label was present in erythroblasts [ **A**], primitive erythrosomes like the one separating from the cell that made it (left lower corner in [ **B**]), platelets [ **C**] and in EC [ **D**]. Morphologically interesting cells, erythroblast [ **E**] and perishing hemangioblast [ **F**], stained with nonspecific antibody are shown at low magnification (they had no gold grains). The erythroblast was entirely consumed by a successful incremental conversion into at least three erythropoietic vesicles [ **E**] and the cell with ambivalent phenotype [ **F**], possibly hemangioblast, contained three internal vesicles resembling erythrosomes and two of comparable size that appeared to be failing as erythrosomes (partially electron lucent ones).

**Figure S5.**
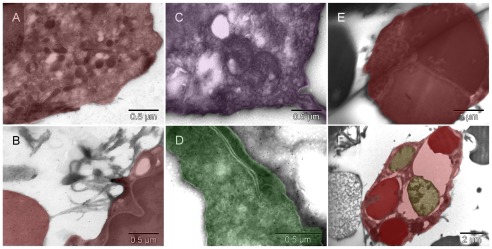
Cells of graft origin labeled with GFP under ubiquitin promoter. GFP-specific label was present in erythroblasts (red) [ **A**], primitive erythrosomes (red) like the one separating from the cell that made it (left lower corner in [ **B**]), platelets (purple) [ **C**] and in EC (green) [ **D**]. Morphologically interesting cells, erythroblast [ **E**] and perishing hemangioblast [ **F**] stained with nonspecific antibody are shown at low magnification (they had no gold grains). The erythroblast was entirely consumed by a successful incremental conversion into at least three erythrogenic vesicles (red) [ **E**] and the cell with ambivalent phenotype [ **F**], possibly hemangioblast, contained three internal vesicles resembling erythrosomes (red) and two of comparable size that appeared to be failing as erythrosomes (green) (partially electron lucent ones).

Detecting the green fluorescent protein (GFP) label of either the graft or the tumor spheroids helped to address the question of the source of hemato-vasculogenic cells in the tumor microenvironment. In Mouse 5, the graft tissue originated from the donor mouse that carried the GFP marker under a ubiquitin promoter. Thus the GFP label found in erythroblasts, erythrosomes, platelets and ECs indicated participation of the graft cells in building of the tumor vasculature ( Figure 5 & Figure S5), although unlabeled cells with similar morphology were also present. Cells with ambivalent morphology, like the one in Figure 5 & Figure S5 [F], provided a hint that ECs were evolving from erythroblasts.

**Figure 6.**
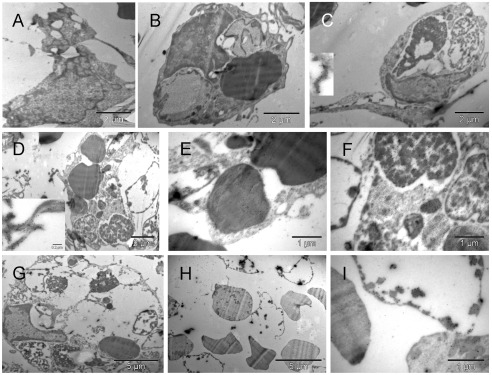
HSCs differentiation by evolution. The cells shown in the upper row [ **A**, **B** & **C**] were located in the hyaline membrane 5 µm (or less) apart from one another ( Figure 7 & Figure S7) suggesting their relatively recent derivation from a common precursor. The first, irregularly shaped cell with platelet-resembling attachment was probably a megakaryocyte [ **A**]. The second one, had four large vesicles but only one of them was similar in size and electron density to a cell-free erythrosome (right upper corner in [ **A**] and elswhere), whereas two other vesicles were much lighter with the exception of small patches at the periphery, and the fourth one was probably an incompletely converted nucleus [ **B**]. The third cell had two large vesicles and both appeared to represent different stages of degradation of the material they were made of [ **C**]. The outer membrane of that cell was at one side connected to another cell via calmyrin-containing extracellular fibers (insert in [ **C**]). Both cells with the large vesicles made impression of cells with mixed phenotypes with some inclinations to specialize in either producing erythrosomes [ **B**] or forming a lumen [ **C**]. Fields shown in [ **D**– **I**] were in the same section; they represented what seemed to be left after internal remodeling of polyploidal cells or multi-cellular clusters [ **D** & **G**]. There were cell-free erythrosomes as well as empty vesicles, some broken [ **H**]. Clearly, cells were perishing because of such restructuring but they were also changing the microenvironment. Insert in [ **D**] shows calmyrin-specific label on fibers of ECM. [ **E**, **F** & **I**] are enlarged regions from [ **D**] and [ **H**], respectively.

**Figure 7.**
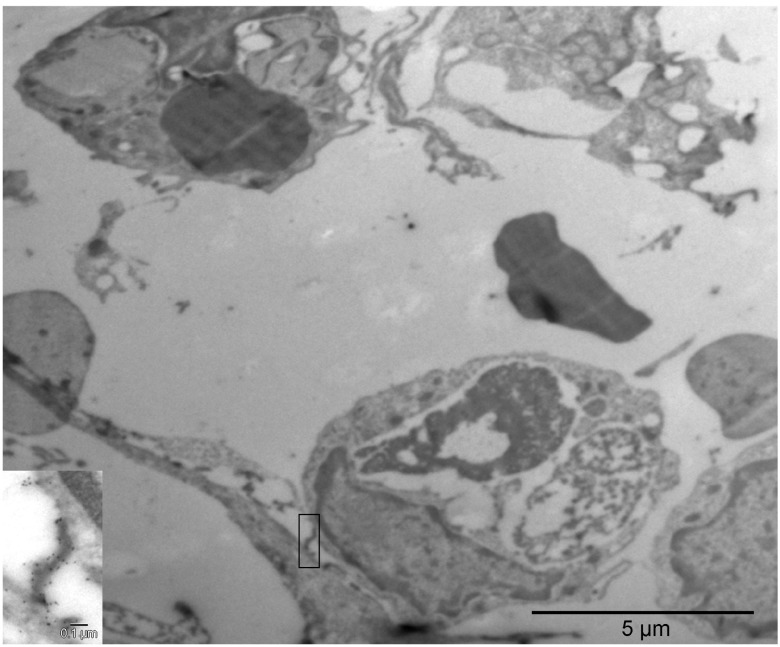
Relative location of megakaryocyte, erythroblast and ECs precursors *in situ*. Compare with Figure 6 [A, B & C]. The insert shows calmyrin-containing extracellular fibers connecting the adjacent cells.

**Figure S6.**
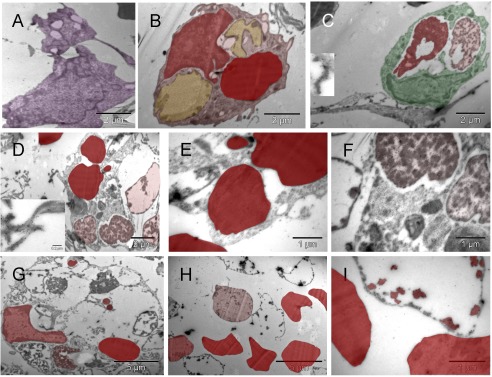
HSCs differentiation by evolution. The cells shown in the upper row [ **A**, **B** & **C**] were located in the hyaline membrane 5 µm (or less) apart from one another ( Figure S14 b) suggesting their relatively recent derivation from a common precursor. The first, irregularly shaped cell with platelet-resembling attachment was probably a megakaryocyte (purple) [ **A**]. The second one (mixed shades of red), had four large vesicles but only one of them was similar in size and electron density to a cell-free erythrosome (right upper corner in [ **A**]), whereas two other vesicles (yellow) were much lighter with the exception of small patches at the periphery, and the fourth one was probably an incompletely converted nucleus [ **B**]. The third cell (green) had two large vesicles (red and pink) and both appeared to represent different stages of degradation of the material they were made of [ **C**]. The outer membrane of that cell was at one side connected to another cell via calmyrin-containing extracellular fibers (insert in [ **C**]). Both cells with the large vesicles made impression of cells with mixed phenotypes with some inclinations to specialize in either producing erythrosomes [ **B**] or forming a lumen [ **C**]. Fields shown in [ **D**– **I**] were in the same section; they represented what seemed to be left after internal remodeling of polyploidal cells or multi-cellular clusters [ **D** & **G**]. There were cell-free erythrosomes as well as empty vesicles, some broken [ **H**]. Clearly, cells were perishing because of such restructuring but they were also changing the microenvironment. Insert in [ **D**] shows calmyrin-specific label on fibers of ECM. [ **E**, **F** & **I**] are enlarged regions from [ **D**] and [ **H**], respectively.

**Figure S7.**
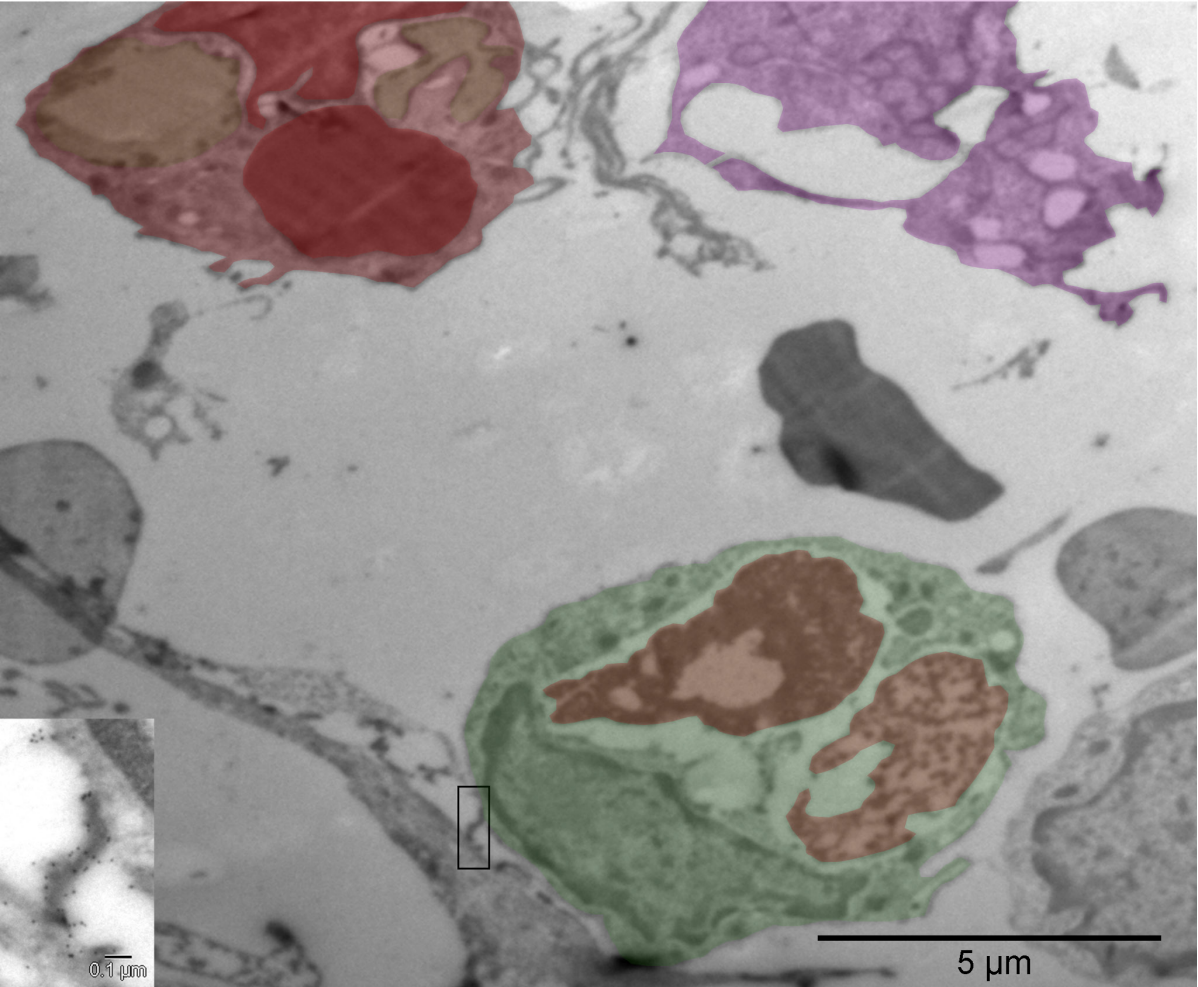
Relative location of megakaryocyte (purple), erythroblast (red) and ECs (green) precursors *in situ*. Compare with Figure S4 [A, B & C]. The insert shows calmyrin-containing extracellular fibers connecting the adjacent cells.

We wondered if a mechanism similar to the one controlling hemostasis could play a role in establishing the vascular pattern before forming the endothelium. A protein best known for its involvement in platelet aggregation and thrombus stabilization, calmyrin, also known as calcium and integrin-binding protein (CIB1), was therefore detected next. Consequently, a new role for calmyrin in tissue morphogenesis emerged from such analysis of the hyaline membrane where the cells were mostly separated from one another. At low magnification, occasionally triplets of cells were seen within a single field; each one with different features that suggested that they were related but currently committing to become either megakaryocytes, erythroblasts or ECs ( Figure 6 & Figure S6 [A, B & C] and Figure 7 & Figure S7). The calmyrin-specific label was located in all three morphologically distinct cells and in the fibers connecting the differentiating ECs with another cell and in the ECM ( Figure 6 & Figure S6 [C & D] respectively), as well as in large, possibly polyploidal cells that contained internal vesicles similar to those in the cells of regular size ( Figure 6 & Figure S6 [D & G]). Those vesicles were either becoming erythrosomes or losing their electron-dense content. The cells were perishing in the process but at the same time, they were changing the microenvironment by leaving traces of their presence in the ECM as developmental clues for other cells. All that suggested that the differentiation process was happening there by means of trial and error, i.e. cytoevolution. Calmyrin seemed to be instrumental in building this tissue canvas. Overall distribution of the protein inside the cells was compartmentalized ( Figure 6 & Figure S6 [E, F & I]) but within each compartment, the calmyrin label was dispersed randomly. Inside the primitive erythrosomes of the hyaline membrane, the label was also dispersed (with rare exceptions), unlike in those from the solid tissue closer to the tumor.

**Figure 8.**
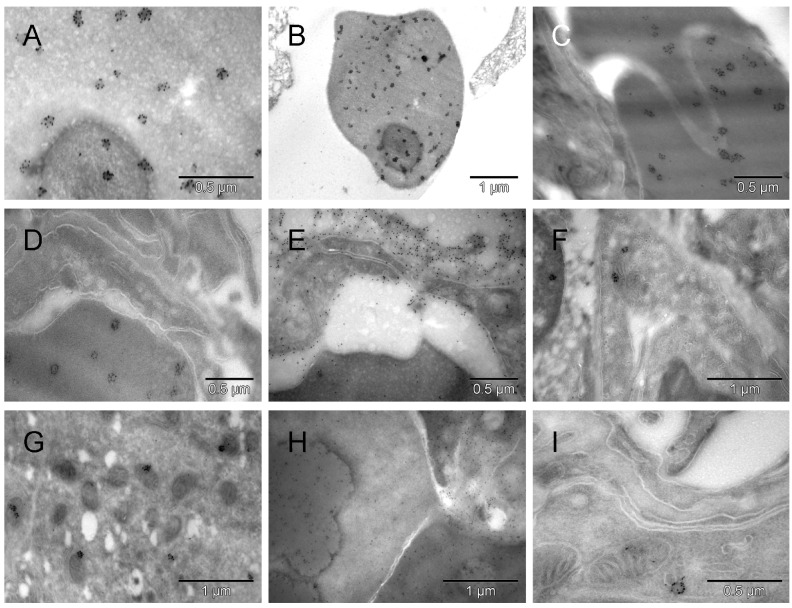
Calmyrite: a new ultrastructural entity involved in anaerobic metabolism. Uneven distribution of the abundant calmyrin-specific label in erythrosomes from the solid tissue under the hyaline membrane was striking [ **A**, **B**, **C** & **D**]. No such clusters were seen extracellularly [ **E**]. The gold particles appeared arranged with certain symmetry in clusters of about 6 to 12, as if the protein became a part of distinct para-crystalline ultrastructures that in less differentiated regions were not formed ([ **E**] and Figure 6 & Figure S6). Such gold grain clusters were also present in ECs [ **F**] and tumor cells [ **G**] where small dark mitochondria suggested hypoxic distress. In a giant cell the uneven distribution of scattered calmyrin-specific label and heterogeneity of electron density indicated an ongoing process of erythrogenic conversion [ **H**]. One calmyrite in a section from normal rat lung is shown in [ **I**]. Those ultrastructural para-crystalline elements visualized by calmyrin-specific label are being described here for the first time and referred to as calmyrites.

**Figure S8.**
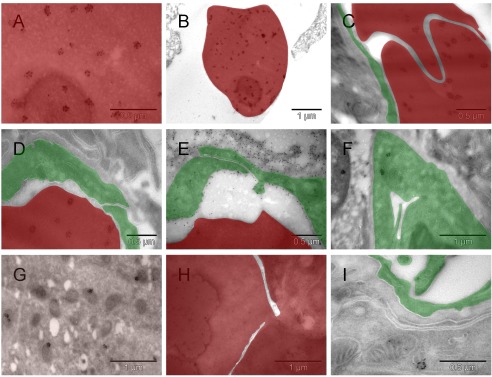
Calmyrite: a new ultrastructural entity involved in anaerobic metabolism. Uneven distribution of the abundant calmyrin-specific label in erythrosomes (red) from the solid tissue under the hyaline membrane was striking [ **A**, **B**, **C** & **D**]. No such clusters were seen extracellularly [ **E**]. The gold particles appeared arranged with certain symmetry in clusters of about 6 to 12, as if the protein became a part of distinct para-crystalline ultrastructures that in less differentiated regions were not formed ([ **E**] and Figure S4). Such gold grain clusters were also present in ECs (green) [ **F**] and tumor cells (brown) [ **G**] where small dark mitochondria suggested hypoxic distress. In a giant cell, the uneven distribution of scattered calmyrin-specific label and heterogeneity of electron density indicated an ongoing process of erythrogenic conversion [ **H**]. One calmyrite in a section from normal rat lung is shown in [ **I**]. Those ultrastructural para-crystalline elements visualized by calmyrin-specific label are being described here for the first time and referred to as calmyrites.

In those more differentiated cells beneath the hyaline membrane, the calmyrin-specific label formed clusters over an area of about caveolar size, i.e. 70 nm in diameter ( Figure 8 & Figure S8) revealing a new ultrastructural element, the calmyrite. The sub-cellular location of the calmyrites in mitochondria, peroxisomes and erythrosomes, suggested that all three of those organelles were homing previously unreported ultrastructural elements related to energy metabolism. In addition to cell types shown in Figure 8 & Figure S8 [A–H], calmyrites were seen in megakaryocytes, platelets, granulocytes, plasma cells, smooth muscle and rat normal EC(s). The included rat lung section ( Figure 8 [I]) demonstrated that calmyrites existed in normal differentiated cells together with mitochondria and were not a product of necrotic process, but rather representing a backup metabolic pathway rendering robustness to the cellular metabolism.

**Figure 9.**
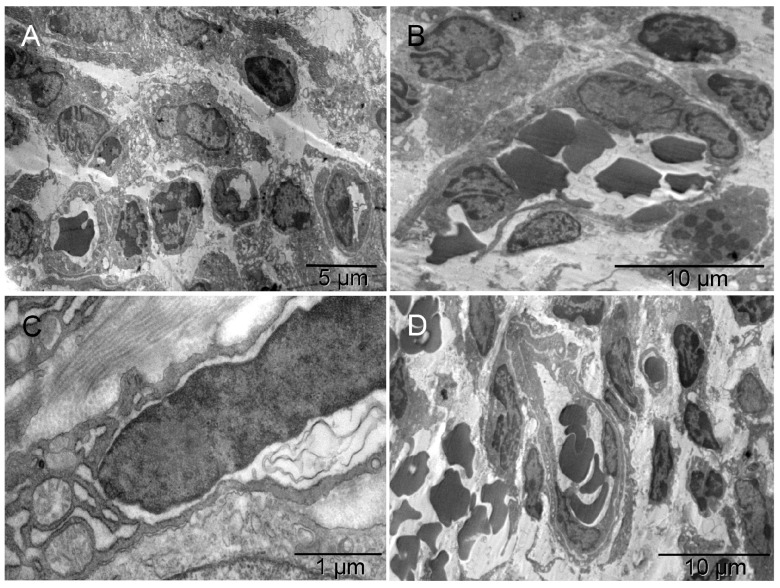
Progress in cellular differentiation and assembly of new vessels. Three weeks after implantation, erythroblasts and megakaryocytes were accompanied by capillaries [ **A**]. The close proximity of those three cell types suggested their recent differentiation from common precursors (HSCs) at that location [ **A**]. Also evident were forming vessels larger than capillaries, i.e. nucleated cells extending themselves around groups of erythrosomes before completing the enclosure [ **B**]. The snail shaped cell in the upper left corner of [ **B**] had asymmetrically distributed cytoplasm (a feature of a megakaryocyte) flattened against the abluminal surface of the forming vessel like a podosome, and nucleus partially divided by a radial sinus or invagination of nuclear membrane, suggestive of a potential for stretching. Cells closer to the erythrosomes also had such features. A cell undergoing mitosis is in the lower right corner of the image. Some cells, with ambivalent phenotype that had not specialized into either erythrosome or EC, made collagen in exuberant amounts, comparable to the size of their entire cytoplasm (instead of a few fibers like in Mouse 5) and the nucleus seemed to be converting into an erythrosome on its own [ **C**]. However, because those proficient collagen makers were also perishing in the process of tissue building, the result was an area filled with scattered erythrosomes amidst massive amounts of collagen like in the left lower corner in [ **D**]. Capillaries and larger vessels were seen side by side [ **D**].

**Figure 10.**
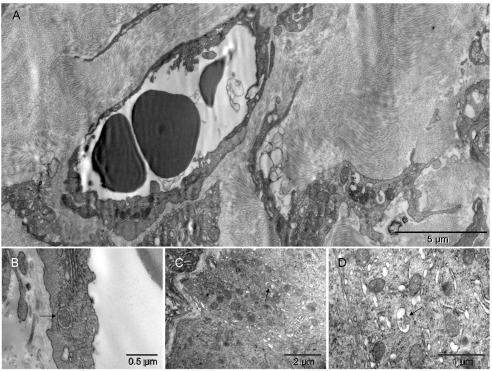
Role of collagen in morphogenesis of primitive vessels. The primitive vessels were embedded in copious amounts of collagen [ **A**]. The wall of such vessels typically was polarized; it had caveolae on the luminal surface and collagen abluminally [ **B**]. Collagen-making cell and tumor edges, accommodating each other’s curvatures, revealed interaction between the two [ **C**]. The tumor seemed to benefit from collagen support provided by the nonmalignant neighbor as well as appearingto have acquired the ability to synthesize some by itself. Arrows in [ **B**, **C** & **D**] point towards intracellular bundles of collagen fibers; [ **D**] is the enlarged collagen-containing region of [ **C**]. Dilated cisternae of endoplasmic reticulum depleted of ribosomes to a variable degree resembled vesiculo-vacuolar organelles (VVOs) ^59^ [ **D**].

**Figure S9.**
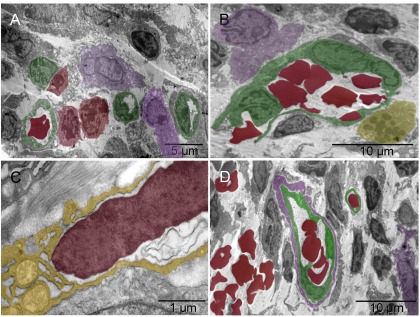
Progress in cellular differentiation and assembly of new vessels. Three weeks after implantation, erythroblasts (red) and megakaryocytes (purple) were accompanied by capillaries (green) [ **A**]. The close proximity of those three cell types suggested their recent differentiation from common precursors (HSCs) at that location [ **A**]. Also evident were forming vessels larger than capillaries, i.e. nucleated cells extending themselves around groups of erythrosomes before completing the enclosure (green) [ **B**]. The snail shaped cell (purple) in the upper left corner of [ **B**] had asymmetrically distributed cytoplasm (a feature of a megakaryocyte) flattened against the abluminal surface of the forming vessel like a podosome, and nucleus partially divided by a radial sinus or invagination of nuclear membrane, suggestive of a potential for stretching. Cells closer to the erythrosomes also had such features. A cell undergoing mitosis (bright yellow) is in the lower right corner of the image. Some cells, with ambivalent phenotype that had not specialized into either erythrosome or EC, made collagen in exuberant amounts, comparable to the size of their entire cytoplasm (yellow) instead of a few fibers like in Mouse 5, and the nucleus (red) seemed to be converting into an erythrosome on its own [ **C**]. However, because those proficient collagen makers were also perishing in the process of tissue building, the result was an area filled with scattered erythrosomes amidst massive amounts of collagen like in the left lower corner in [ **D**]. Capillaries and larger vessels were seen side by side [ **D**].

**Figure S10.**
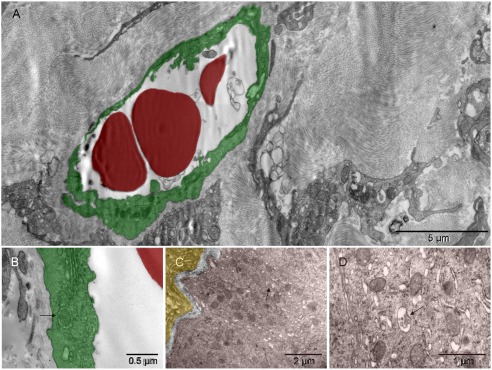
Role of collagen in morphogenesis of primitive vessels. The primitive vessels were embedded in copious amounts of collagen [ **A**]. The wall of such vessels (green) typically was polarized; it had caveolae on the luminal surface and collagen abluminally [ **B**]. Collagen-making cell (yellow) and tumor edges (brown), accommodating each other’s curvatures, revealed interaction between the two [ **C**]. The tumor seemed to benefit from collagen support provided by the nonmalignant neighbor as well as appearing to have acquired the ability to synthesize some by itself. Arrows in [ **B**, **C** & **D**] point towards intracellular bundles of collagen fibers; [ **D**] is the enlarged collagen-containing region of [ **C**]. Dilated cisternae of endoplasmic reticulum depleted of ribosomes to a variable degree resembled vesiculo-vacuolar organelles (VVOs) ^59^ [ **D**].

Three weeks after implantation, some cells kept converting into the erythrosomes and others kept providing structural support to them but in a more complex way. Their phenotypes kept changing ( Figure 9 & Figure S9). Some cells were producing large amounts of collagen as if converting their entire cytoplasm into masses of collagen while their nuclei were converting into erythroschizosomes ( Figure 9 & Figure S9 [C]) leading to a new tissue canvas in the form of fields of collagen with erythrosomes scattered in there. Such vessel-free erythrosomes described earlier in a similar context were believed to be extravasated erythrocytes. Eosinophils, adipocytes and dendritic cells were occasionally present in that environment as well. Erythrosomes and cells providing structural support to them seemed to have been chemotacticly attracted to each other regardless of the current level of their differentiation. Moreover, segments of new vasculature with increasing structural complexity were assembling around emerging erythrosomes into capillaries and vessels of variable size simultaneously. Occasionally, megakaryocyte-resembling cells were positioning themselves like pericytes, i.e. abluminally, close to a forming vessel. The one in Figure 9 & Figure S9 [B] might have been a megakaryocyte evolving into ECs or pericyte. Still greater complexity of the vasculature morphogenesis was evident in the second mouse with tumor grown for three weeks. D epending on the location in the section, the more primitive forms of the vasculature were present as well. Collagen came across as an important factor for building not only vasculature but also tumor tissue ( Figure 10 & Figure S10). Signs of hypoxic stress were in the form of relatively small darkening or necrotic mitochondria (loosing their internal structure) and dilated cisternae of endoplasmic reticulum (ER) partially depleted of ribosomes and resembling vesiculo-vacuolar organelles (VVOs) ^59^, ( Figure 10 & Figure S10 [D]).

**Figure 11.**
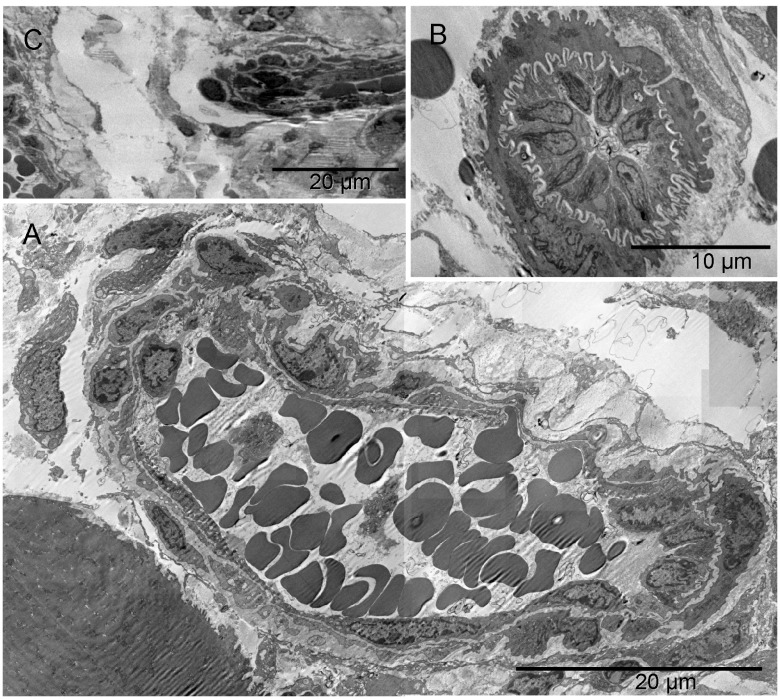
Morphogenesis of an artery (I). Forming lumen of the emerging vasculature fragment shown in [ **A**] was occupied by free erythrosomes and platelets. That “vasculature piece” was not a vessel yet because it included blood elements that were being generated simultaneously with the vessel wall. The wall was incomplete at the time of image capturing. Structural differences along the circumference of the future lumen included a gap on the left side. Such lack of continuity and the irregular forms of erythrosomes also indicated that the forming vessel was not yet a part of the circulation. In that region, cells other than endothelial cells were recognizable, namely chromaffin cells and megakaryocytes at the outer side although their products (platelets) accompanied the erythrosomes in the lumen. The right side of the vessel wall was structurally the most advanced; it consisted of ECs uncharacteristically non-flattened. The shape of their nuclei and corrugation of the basement membrane between those cells and supporting pericyte suggested a potential for physical expansion. Such wavy basement membrane likely contained elastic fibers. The image in [ **B**] could have been from a similar region cut in a perpendicular plane with regard to that in [ **A**]; nuclei of the centrally located ECs had their radial sinuses oriented towards the basement membrane. That too could facilitate the expansion of the vessel. ECs in the middle part of the main image [ **A**] were flattened; in the upper side one could see an erythrosome passing through. The spatial relationship between structures shown in [ **A**] and in Figure 13 is demonstrated in [ **C**].

**Figure 12.**
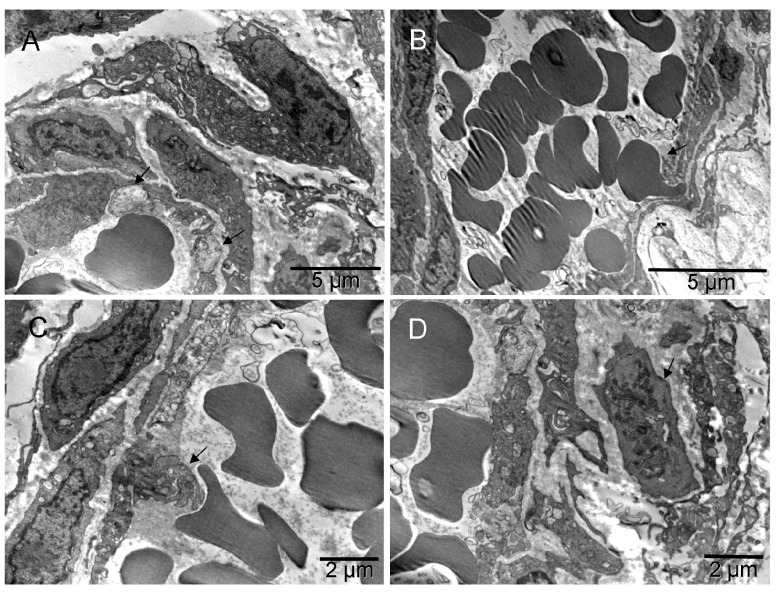
Ongoing structural rearrangements in the less advanced region of the forming artery (I). The images were from a section parallel to the one shown in Figure 11, i.e. from another region of the same vessel. Arrows point to two chromaffin cells in [ **A**]; erythrosome, platelets and maturing platelets *“ante portem”*, i.e. facing opening in the endothelium, are shown in [ **B**, **C** & **D**], respectively.

**Figure 13.**
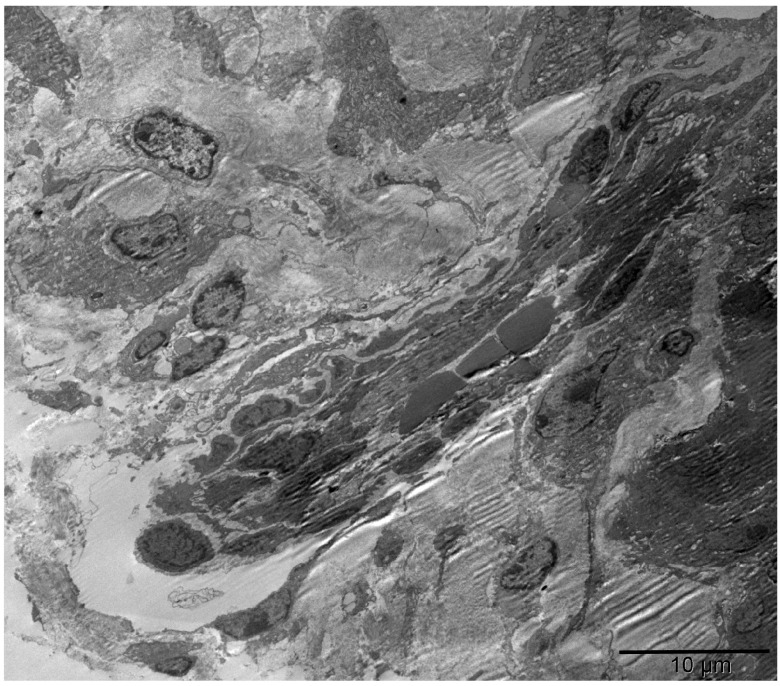
Morphogenesis of an artery (II). A second forming complex vessel had a corrugated basement membrane at both ends of the elongated structure suggesting that the one in Figure 11 might not have been cut exactly parallel to its long axis. A round cell surrounded by a collagen-free region at the lower end suggested growth in that direction which happened to be pointing towards the first artery Figure 11 [C].

**Figure S11.**
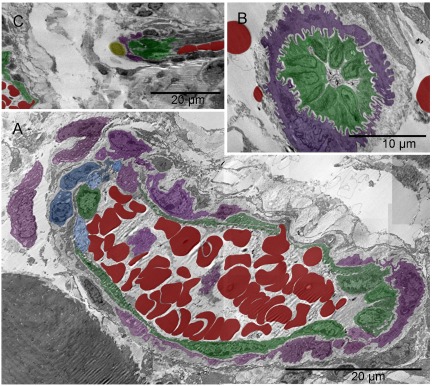
Morphogenesis of an artery (I). Forming lumen of the emerging vasculature fragment shown in [ **A**] was occupied by free erythrosomes (red) and platelets (purple). That “vasculature piece” was not a vessel yet because it included blood elements that were being generated simultaneously with the vessel wall. The wall was incomplete at the time of image capturing. Structural differences along the circumference of the future lumen included a gap on the left side. Such lack of continuity and the irregular forms of erythrosomes also indicated that the forming vessel was not yet a part of the circulation. In that region, cells other than endothelial cells were recognizable, namely nerve and chromaffin cells (dark and light blue, respectively) and megakaryocytes (purple) at the outer side; although their products, platelets (purple), accompanied the erythrosomes in the lumen. The right side of the vessel wall was structurally the most advanced; it consisted of ECs (green) uncharacteristically non-flattened. The shape of their nuclei and corrugation of the basement membrane between those cells and supporting pericyte suggested a potential for physical expansion. Such wavy basement membrane likely contained elastic fibers. The image in [ **B**] could have been from a similar region cut in a perpendicular plane with regard to that in [ **A**]; nuclei of the centrally located ECs had their radial sinuses oriented towards the basement membrane. That too could facilitate the expansion of the vessel. ECs in the middle part of the main image [ **A**] were flattened; in the upper side one could see an erythrosome passing through. The spatial relationship between the structures shown in [ **A**] and in Figure S10 is demonstrated in [ **C**].

**Figure S12.**
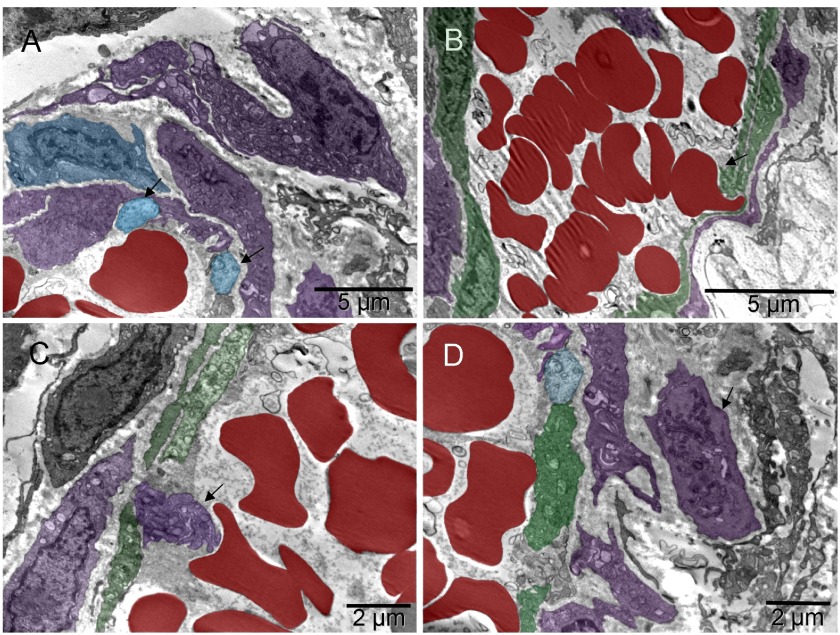
Ongoing structural rearrangements in the less advanced region of the forming artery (I). The images were from a section parallel to the one shown in Figure S8, i.e., from another region of the same vessel. Arrows point to two chromaffin cells (blue) in [ **A**]; erythrosome (red), platelets (purple) and maturing platelets “ante portem” i.e. facing the opening in the endothelium (green), are shown in [ **B**, **C** & **D**], respectively.

**Figure S13.**
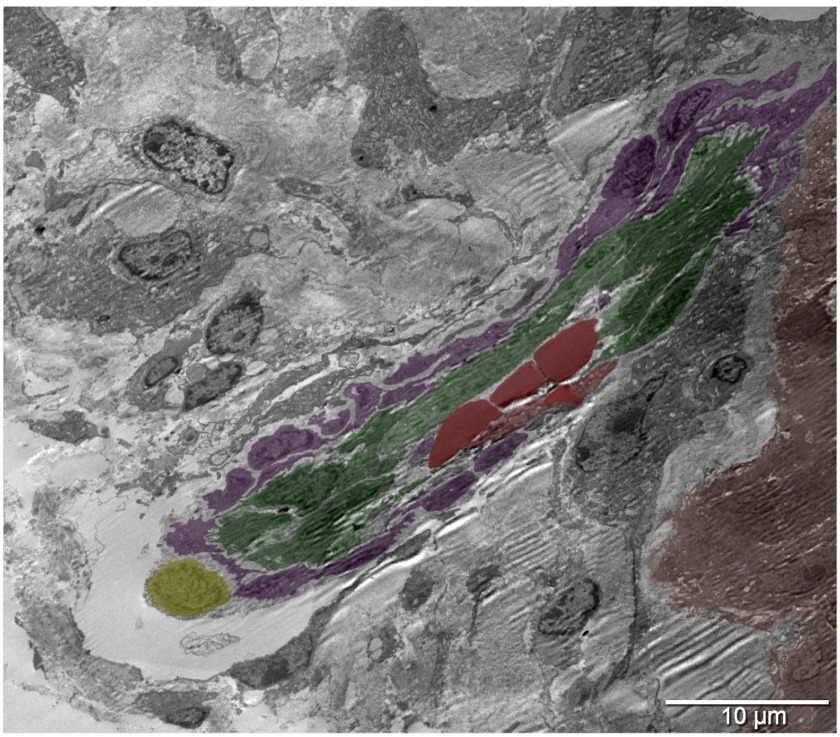
Morphogenesis of an artery (II). A second forming complex vessel had a corrugated basement membrane at both ends of the elongated structure suggesting that the one in Figure S8 might have been cut not exactly parallel to its long axis. A round cell (bright yellow) surrounded by a collagen-free region at the lower end suggested growth in that direction which happened to be pointing towards the first artery Figure S8 [C].

Arteries and veins were formed independently, each vessel type from individual, differentiated cells. The level of differentiation varied. The two shown arteries assembling from differentiated cells of variable type (including chromaffin cells) would have likely joined eventually had the tissue been harvested later ( Figure 11, Figure 12, Figure 13 & Figure S11, Figure S12, Figure S13).

**Figure 14.**
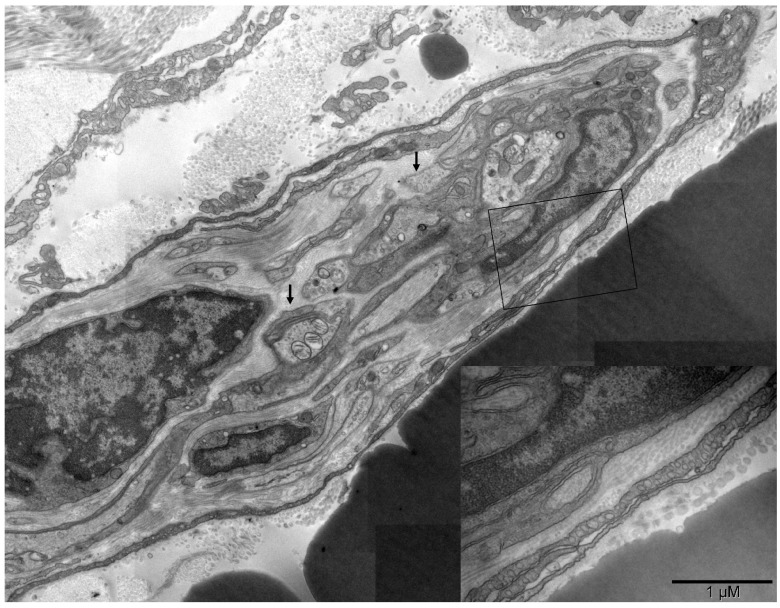
Developing peripheral nerve. Several nerve fibers were engulfed by Schwann cells on the right but progressing myelination could be recognized only on the one in the center with three mitochondria in the section plane (left arrow). Multiple nerve boutons with synaptic vesicles (right arrow) were present but mature synapses were absent. Surrounding the nerve fibers was one to three-layer neurothelium with abundant caveolae (insert in the right lower corner). Collagen fibers located internally were thinner (20–40 nm) than those lining the external surface of the neurothelium (60–100 nm). A giant erythroschizosome was located next to the forming nerve and it had similar dimensions (right lower corner behind the insert).

**Figure 15.**
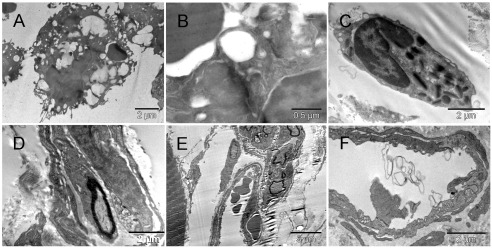
Rare findings. [ **A** & **B**]: Erythrosome labeled with ubiquitin-detecting antibody, internalized by non-labeled macrophage (Mouse 5). [ **C**]: Eosinophil (Mouse 3). [ **D**]: Myelinated nerve fiber engulfed by Schwann cell (Mouse 2). [ **E**]: Forming vessel located between muscle and nerves (Mouse 2). [ **F**]: Empty vein or lymphatic vessel with an internal valve (Mouse 2).

**Figure S14.**
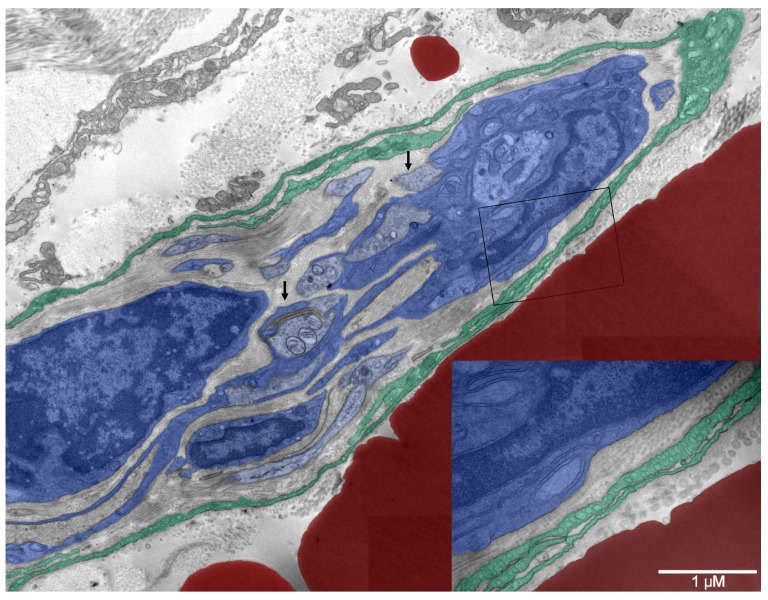
Developing peripheral nerve. Several nerve fibers (light blue) were engulfed by Schwann cells (dark blue) on the right but progressing myelination could be recognized only on the one in the center with three mitochondria in the section plane (left arrow). Multiple nerve boutons with synaptic vesicles (right arrow) were present but mature synapses were absent. Surrounding the nerve fibers was one to three-layer neurothelium (emerald green) with abundant caveolae (insert in the right lower corner). Collagen fibers located internally were thinner (20–40 nm) than those lining the external surface of the neurothelium (60–100 nm). A giant erythroschizosome (red) was located next to the forming nerve and it had similar dimensions (right lower corner behind the insert).

**Figure S15.**
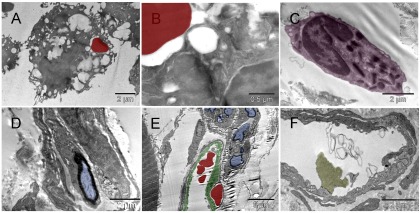
Rare findings. [ **A** & **B**] Erythrosome labeled with ubiquitin-detecting antibody, internalized by non-labeled macrophage (Mouse 5). [ **C**] Eosinophil (Mouse 3). [ **D**]: Myelinated nerve fiber (blue) engulfed by Schwann cell (Mouse 2). [ **E**] Forming vessel (green) located between muscle and nerves (Mouse 2). [ **F**] Empty vein or lymphatic vessel with an internal valve (olive green) (Mouse 2).

Moreover, developing nerves accompanied some forming ves­sels ( Figure 14, Figure S14, and Figure 15 & Figure S15 [D & E]). One displayed a few nerve fibers at early stages of my­elination and a lack of mature synapses despite the presence of multiple nerve boutons with synaptic vesicles. Next to it, an equivalent number of cells committed to the erythropoietic pathway generated a giant erythro-schizosome, although perhaps normally there would have been a cluster of erythrosomes ("erythropoietic lake") in a forming vessel. Whether this particular one was going to be successful in producing normal erythrosomes or was just a part of the ongoing cytoevolution was not clear. The cells that surrounded the developing nerve did not necessarily derive from the ectoderm. They ended up on the surface of neuroplax but had much in common with endothelium, morphologically and functionally – namely, a flattened shape, plenty of caveolae to control the microenvironment of the developing neurons and Schwann cells. Caveolae were shown elsewhere to contain ATP-ase that could be processing ATP secreted by the giant erythroschizont right next to it ^60– 63^. Such concerted differentiation resulting in different functions would have been unlikely to happen without coordinated regulation of the vasculature and nerves.

In summary, the observed variants of erythrogenesis included nucleo-cytoplasmic conversion and nuclear conversion with collagen production continuing in the cytoplasm. Erythroblasts were either entirely devoted to erythrogenesis or had ambiguous phenotype, including electron lucent (failing) vesicles, collagen synthesis and later also presence of caveolae. Erythrogenic conversion affected entire cells or syncytium and later generated schizomers (myeloplaxes, cobble stone areas, erythrocytic lakes) or the cellular remodeling occurred incrementally. Erythroblasts had aerobic or anaerobic metabolism as indicated by the presence or absence of mitochondria. They were polynuclear or mononuclear and of giant or average cell size. The erythrosomal membrane was or was not undergoing remodeling inside the erythroblast before release of the organelle. Some cells were perishing without succeeding in production of either proper erythrosomes or a lumen despite initiating those processes. Evidently, the cellular differentiation was happening by means of natural selection (cytoevolution). Cells perishing in the process were contributing to building tissue framework available for subsequent remodeling. Erythropoiesis appeared to be essential at every step of evolving cellular mechanisms of vasculature formation. Fusion of the neo-vasculature with the pre-existing one would be the final step needed to allow the newly created portion of tumor tissue, already equipped with its own vasculature (a network of blood-containing vessels of variable size), to grow beyond the limit set by the diffusion range of metabolites. That event would be equivalent to the first heartbeat in a developing embryo.

## Discussion

### Cancer stem cells

Judging by the activities of cells evolving in our new experimentally created environment, we retrospectively assumed the presence of their precursors, in agreement with the functional TSCs definition. The question was which cells participated in the formation of the tumor vasculature and how did the vasculature form? At the outset, the components of the tumor niche (tumor spheroid, graft and local host tissues) did not have an ongoing vasculature formation. In both variants of the model, with and without homologous tissue graft, the new qualities emerged after the implantation (this report and the accompanying article ^43^, respectively). New tissues formed locally and integrated into the host animal. Some of the graft cells formed the new vasculature for the pseudo-orthotopically implanted tumor, whereas in the absence of the graft, some of the tumor cells eventually evolved to do the same. In both types of the tumor niche (pseudo-orthotopic and ectopic) some local cells differentiated to form vasculature at the site of the tissue growth. Regardless of the origin (lineage) of those cells, one could think of them as SCs - based on the definition of such cells ^37^.

The amorphous continuously growing tumor mass was out of touch with tissue morphogenesis-controlling mechanisms but depended on the ability of the tumor cells to induce formation of vasculature either by CSCs or by local TSCs. Thus, tumors were subjected to selective pressure because those incapable of either inducing local nonmalignant SCs to form tumor-supporting vasculature or of generating their own vasculature ^43^ could not grow. The ability of the tumor to generate its own vasculature in the ectopic environment implied a conditional existence of CSCs, i.e. evident in one *in vivo* environment but not in the other (and not *in vitro*). Any single cell of the spheroid population could potentially establish new tumors in the orthotopic environment. However, large numbers of those cells would be necessary in a variety of ectopic environments, whether encountered by metastatic tumors or created experimentally ^43^. One should not be surprised at finding HSCs markers among CSCs and ESCs because some of the features perceived in the literature as defining the stemness might be attributed to tissue growth, shared by malignant and non-malignant tissues. Our results showed that growing tissue sectors were capable of generating their own vasculature (blood and vessels) independently of the existing circulatory system and therefore recapitulated the embryonal vasculogenesis. However, the newly created blood-containing vascular segments did eventually fuse with the host vasculature whereas the embryonal vasculature does not fuse with the maternal one.

The vascular "sprouts" grew towards existing vessels, not away from them ( Figure 11 & Figure S11 [A, C] and Figure 13 & Figure S13). Yet the new tumor-supporting vasculature observed here originated from the graft, not the tumor. To understand the paradox, one needs to keep in mind that the implanted components, tumor spheroid and graft, were related; both originated from the breast. When implanted separately, each behaved differently. The graft (injured lactating breast tissue) first produced new normal mammary epithelial ducts (results not shown) whereas the tumor initially used some of its own cells to feed the others, a fraction of which evolved into megakaryocytes and ECs ^43^. However, when implanted together, breast tumor and breast tissue reconstituted the primary tumor in its native environment, as intended. Self-organizing potentials of the two influenced each other and a new dynamic balance emerged because of such interaction. At the same time, the host was responding to the injury inflicted by the surgical procedure associated with the implantation. That enabled the comparison of the tumor and the scar tissue vasculature formation side-by-side. The involvement of non-malignant TSCs in the formation of the tumor vasculature suggested that normal and malignant tissues share the cellular mechanism of vasculature formation. The developing nerves accompanying the forming vessels also indicated that those vessels were normal, yet they were as close to a muscle as to the tumor ( Figure 11 & Figure S11). The growing undersupplied region likely initiated the process because without new substrates, the growth would be energetically prohibitive, regardless of the genetic makeup. The oxygen diffusion range and the maturity level of cells in the microenvironment shaped the initial vascular pattern. The presence of calmyrites in non-malignant cells suggested that anaerobic metabolism was not unique for tumors either but could function in normal cells as an alternative, less-efficient pathway functional during mitosis and therefore more frequently used by tumors ^43^. Morphologically, the tumor vascular pattern appeared abnormal because it never matured due to perpetual tumor growth ^64^. Vascular tortuosity had been noticed to be a feature of young tissues ^65, 66^, presumably in anticipation of growth. The blood flow would help to establish the hierarchical pattern for optimal functionality.

### HSC lineages

Traditionally, the ontogenetic tree symbolizing cellular differentiation pathways, for instance hematopoiesis, consisted of arrows arranged in certain sequence and often branching but always pointing in the same direction, therefore signifying progress and irreversibility. Such a concept of phenotypic changes does not reflect great flexibility in terms of the functions of differentiated cells for which compelling evidence has already been accumulated, including *in vitro*-induced pluripotent SCs ^67, 68^, and is fast growing as the stem cell research prospers. The reliance of erythroblast and EC differentiation on natural selection, i.e. differentiation of HSCs by cytoevolution illustrated here for the first time, suggested "broken lines" for lineages (the dead ends and some stage skipping). The process was characterized by great variability of forms and perishing of nonfunctional ones that nevertheless must have contributed to the progress in differentiation of others by changing the microenvironment. The dead ends were demonstrated also *in vitro* ^69^. Another interesting point related to cell lineages that emerged here as examples of the cell types thought of as mature that could trans-differentiate into other phenotypes. Megakaryocytes could become pericytes or ECs, depending on circumstances, and collagen-producers could become ECs. To trans-differentiate might be more practical than to migrate long distances and deal with the forms that are no longer useful. The requirement for continuous regulation to maintain a differentiated phenotype was postulated previously ^70^. A network of arrows pointing in different directions, including reverse, would represent the actual cellular differentiation pathways better than the traditional tree. "A good analogy likens cell types to metabolites in metabolic networks" ^40^.

Megakaryocytes are known to develop from bipotent erythroid/megakaryocytic progenitor ^7, 53, 71, 72^. However, their morphogenetic role in establishing vascular pattern in the absence of ECs is new ( Figure 1, Figure 2, Figure 11 & Figure S1, Figure S2, Figure S11). Addressing one of the unanswered questions in vascular biology ^6, 7^ it demystifies the presence of platelets in the early embryo and demonstrates that megakaryocytes and erythroblasts develop before ECs. Together they are capable of forming primitive vascular patterns without ECs. Given the chemical nature of CEA, (200 kDa glycoprotein containing about 50% carbohydrate) and its presence in tumors and in pregnancy, one could suspect that the presumed proteoglycans of ECM ( Figure 1 & Figure S1) indeed contain CEA ^31^. The second dichotomy in the pathway corroborated the controversial derivation of ECs from hemangioblasts ^73^. At a certain phase in vasculature morphogenesis, the three cell types co-localized as triplets and acted in concert. However, as the assembly of vessels progressed, megakaryocytes started positioning themselves abluminally suggesting their ability to trans-differentiate into pericytes ( Figure 9 & Figure S9).

The presence of eosinophils ( Figure 15 & Figure S15), neutrophils ( Figure 1 & Figure S1), dendritic and plasma cells (not shown) in that environment, although relatively rare, suggests that they too differentiate locally and assist tumor growth. That possibility deserves further studies because, if confirmed, it would explain why the host immune system could tolerate the tumors. If primary tumors are similar enough to the tissue of origin to prompt local TSCs for vasculature formation, one could hardly rationalize the need for its rejection by the host. If, however, the tumor loses the tissue-specific characteristics, it would become alienated and dormant until an angiogenic switch occurred ^43, 74^, which would not necessarily be immunogenic (e.g. medical implants are non-immunogenic). In further support of this theory, the lymphopoietic potential of mouse embryonic HSCs cells was indeed observed *in vitro* and *in vivo* ^17, 75^.

### Extramedullar erythrogenesis and tissue morphogenesis

The central role of the extramedullar erythrogenesis at every level of the dynamic complexity of vasculature formation was understandable because the ongoing tissue growth requires energy and nutrients. The complex issue of the origin and maturation of HSCs in developing embryos and their potential migration among AGM, yolk sac, umbilical artery, vitellin artery, placenta and fetal liver ^76^ was echoed in the model used here. Without the guidance from the mature bone marrow niche, the erythrogenesis was recapitulating the embryonal stages. Calmyrites contributed a new criterion for grading the maturity level of erythropoiesis.

Our observations offered new understanding of vasculature morphogenesis in relation to tissue morphogenesis. For example, plasticity of cells became well recognized but often perceived as "puzzling, surprising, intriguing, provoking", etc. as single TSC types or even clones gave rise to multiple phenotypes unexpectedly. Epithelium evolved from bone marrow progenitors ^77^, hematopoietic cells from endothelial or neuronal cells ^75, 78, 79^, blood from brain ^80^ or muscle ^81, 82^, muscle from HSCs ^82, 83^, and HSCs from stromal-vascular fraction of adipose tissue ^21, 84^, causing the confusion. The common presence of the hematopoietic component in those heterogeneous differentiated populations derived from TSCs was striking. It suggests that progenitor cells committed to a specific tissue type also have hematoblastic potential. One can anticipate more reports on finding erythropoietic cells during procedures intended to isolate tissue-specific cells. That reasoning does not contradict trans-differentiation of cell types other than those involved in erythropoiesis. For example, neuronal cells were shown to change their phenotype to endothelial cells when co-cultured with (ECs) ^85^. Historically, the HSC lineage has shaped the traditional belief in terminal differentiation but, as discussed below, that lineage turned out to be a special case rather than the archetype from which to extrapolate to other tissues. Yet supposedly all connective tissues (and possibly others) derive from HSCs and therefore, transplantation of HSCs rather than mesenchymal SCs would be the choice therapy for genetic deficiencies of connective tissues, making HSCs a panacea for multiple related illnesses ^86^. However, our results suggest the opposite. All tissues, at all stages of life, might have hemato-lympho-vaso-neuro-adipo-poietic potentials as long as they have the potential to grow or to repair themselves. However, execution of that potential would depend on morphogenesis-controlling factors and the availability of proper substrates. Various types of hematopoiesis (primitive, definitive and subclasses of those) reflect the differentiation levels of the tissues harboring the process. In an emergency, even endothelium could become hemogenic ^87^.

Understanding the mechanisms regulating tissue morphogenesis could have therapeutic implications. For example, the observation that collagen is essential for the process could be useful. Assuming that ECM of tumor tissue might be unprotected with tissue inhibitors of metalloproteinases (TIMPs), as was shown to be the case in embryo ^88^, one could envision pharmacological intervention with proteolytic enzymes.

### The organoblasts concept

The classic functional definition of the adult TSCs was amended in 2002 to include its flexible and regulated tissue self-organization capability based on cell-cell and cell-environment interactions ^37^. The amended definition allowed for the assumption that TSCs have tissue specificity and the ability to form vasculature *de novo* when needed. Conceptually one could view TSCs as histo-hematoblasts, as we did initially. Just like TSCs and SCs in general, the histo-hematoblasts was a concept, not a clone ^37^. Which cells would produce a new piece of tissue or its vasculature did not have to be predetermined during embryogenesis but later, when and where the growth was to take place. The location of tissue growth must be governed by organogenesis-controlling factors like BMP4 ^89– 91^. An example demonstrating the spatial correlation between hematopoiesis and regions of tissue growth is available for the mouse embryo ^92^. Simply put, when traced back along their ontogenetic lineage, cells engaged in building new vasculature for the growing part of a tissue would not all turn out to belong to a single HSC clone. Such multi-origin does not imply their universal applicability as transplants; as mentioned above, the developmental compatibility was shown to matter to some extent ^18^. Another practical aspect that follows is that a strategy to stop the vasculature formation in tumors should not target specific precursor cells but rather the process itself, regardless of the lineage of cells executing the tumor supporting function. To think of SCs as "some cells" might be helpful. Due to the self-organizing potential of tissues in higher organisms, at any phase of growth and development or life of an individual, some cells in the growing region of an organ might be capable of addressing the basic metabolic needs locally, and of establishing communication with the rest of the organism. Which cells of a given organ they are, may be neither predetermined nor critical but dictated by morphogenesis-regulating factors (malfunctioning in tumors) and local energy metabolism requirements (applicable to tumors).

The concept of histo-hematoblasts was rooted in the fundamental role of neo-hematopoiesis in tissue morphogenesis, namely, fueling metabolism of any tissue mass exceeding diffusion range of oxygen (100 – 200 µm ^93, 94^; 150 µm ^64^) and presumably of nutrients. Neo-hematopoiesis appeared to be the core of the process, both figuratively speaking and literally. Emerging erythrosomes, known for their ability to secrete ATP ^58^, formed the central point to which other cells were chemotacticly attracted and around which endothelium evolved eventually with ATP-ase located in caveolae ^60– 63^. What triggered the local (extramedullar) vasculature formation was the localized incremental tissue growth. Any tissue growth, (developmental, repair-related or malignant) would require new vasculature; whereas, proliferation of cells replacing those that perish while fulfilling their physiological functions would not. Examples are intestinal and skin epithelia, hair follicles and secretory cells of some glands (as discussed in ^87^). One could think of TSCs as "kits" containing potentials for all necessary elements of the tissue to be built. Chromaffin cells participated in assembling the new artery ( Figure 11, Figure S11 & Figure 12, Figure S12) and cells from the immune system were present in that environment too. Developing nerves paralleled the forming vessels of similar dimensions ( Figures 15, Figure S15 [E] and Figure 4 in ^43^). GATA-2 transcription factor required for hematopoiesis was also reported to be involved in the maintenance of the pool of ventral neuronal progenitors ^92^. Moreover, existence of common neural and vascular patterning tools (signaling molecules like ephrins, netrins, slits, semaphorins and neuropilins) was in fact pointed out earlier ^95, 96^. The correlation of vasculature formation with the local development of nerves, mentioned above and elsewhere ^97– 101^, called for modifying the concept of histo-hematoblasts to the organoblasts concept, to cover the observed phenomena more adequately.

The organoblasts (Organo-Bs) is designed to be a generic term for such organ-committed precursor cells. The plural of the term is deliberate here, like in the case of SCs, because those cells neither belong to a unique lineage nor are committed to a specific tissue type within an organ. In particular cases they would be for example: Nephro-Bs (kidneys), Hepato-Bs (liver), Osteo-Bs (bone), Dermo-Bs (skin), Pulmo-Bs (lungs), Entero-Bs (gut), Sarco-Bs (muscle), Spleno-Bs (spleen), Thymo-Bs (thymus), Ovario-Bs (ovary), Testo-Bs or Orchio-Bs (testes), Cerebro-Bs (brain) and Cardio-Bs (heart) and so on. Hemangio-Bs and Neuro-Bs would derive from the organoblasts of each organ independently to form vasculature, lymphatics and peripheral nerves. Accordingly, in the formation of any new tissue (or in the growing segment of an existing tissue), some of the growing cells would become hematoblasts or neuroblasts to connect them to the rest of the given organ in the system. Naturally, such a mechanism of vasculature morphogenesis would result in organ-specific variability in the endothelium. That would be consistent with published reports on the presence of tissue-specific markers on endothelium in particular organs, i.e. functional variability of endothelium in different organs ^102– 107^ as well as ultrastructural differences. Brain vessels provided the most prominent example ^108^. The current definition of TSCs implies their potential to provide differentiated cells specific for a given tissue. The difference between TSCs and organoblasts (or organ SCs) is the ability of the latter to give rise to cells of specific tissue parenchyma as well as all the accessory cells necessary to maintain system homeostasis. All those cells would be locally differentiating from the organoblasts at the site of incremental tissue growth.

From the evolutionary perspective, flexibility is more valuable than rigid perfection of the complex systems. Phylogenetic analysis applied to mouse cell lineages have indicated that extensive cellular mixing homogenized the lineages in the embryo before gastrulation ^41^. Therefore, up to that stage of development, the cells were functionally equivalent. Consequently, dissimilar lineages participated in morphogenesis of particular tissues during organogenesis. Such redundancy combined with cell plasticity and the natural selection at the cellular level might have been compensating for the high number of mutations happening at every mitosis. *C. elegans* can afford to lose an entire individual because one organism can produce ~1000 fertilized eggs, whereas mammals are too complex to take care of mutations that way ^41^.

### Tissue definition

The tissue definition based on the organoblasts concept would consist of two parts: (1) organ location (liver, heart, brain, stomach, pancreas, bone, striated muscle, ovary etc.), and (2) specific function, either unique for that organ, or system integrating (nerves, vasculature and lymphatics), or providing structural integrity. Tissues related to the latter two functions could be similar in different organs but not identical, as known for the endothelial surfaces. [Examples: brain grey matter, brain white matter, brain meninges, brain vessel; gut epithelium, gut smooth muscle, gut vessel; heart artery, heart vein, heart muscle; liver parenchyma, liver vessel; lung epithelium, lung endothelium, lung pulmonary pleura, lung costal pleura; lymph node capsule, lymph node nodule, lymph node hilum; peripheral nerve dendrites, peripheral nerve Schwan cells, peripheral nerve neurothelium; auricular cartilage, bronchial cartilage, articular cartilage, growth plate cartilage; bone marrow, bone cancellous, bone trabecular, bone periosteum, bone capillary; tumor parenchyma, tumor vessel, etc.]. There is more to organ development than differentiation of specific cell types. Dramatic qualitative changes of the tissue fabric in growing organisms occur by tissue remodeling; for example, bone morphogenesis involves formation of properly shaped cartilage first and its subsequent replacement with calcified tissue. In our model, free erythrosomes scattered in abundant collagen formed a scaffold that could support morphogenesis of the malignant tissue or scar.

The position of vasculature as a specific type of connective tissue equal in rank to other differentiated tissues, i.e. created during embryogenesis, deserves to be reassessed. Our results suggest extending the acceptance of the multiple origins of HSC precursors in the embryo to any tissue growth (malignant or reconstructive), also after birth. The vasculature formation (blood and vessels) is unique because it must be a part of any tissue growth, whether developmental, pathological or reconstructive and regardless of age, not just during embryogenesis. The novelty here was that not only vessels but also blood formed *de novo* at the location of growing tissue, not just in bone marrow and spleen. That could presumably occur in any organ. Under stress, a fraction of any tissue could feed the rest of the population if that meant survival of some cells. How the conversion of living cells into a meal could happen would depend on what kind of cells they were and on the microenvironment. Hematopoiesis appeared to have fundamental but accessory role in all tissues. That is why following HSC lineage was not a trivial issue. The implications go beyond cell lineages; they extend to organogenesis.

## References

[1] Vidal M Cusick ME Barabasi AL: Interactome networks and human disease. *Cell.* 2011;144(6):986–998 10.1016/j.cell.2011.02.016 21414488 PMC3102045

[2] Nurse P: The great ideas of biology. *Clin Med.* 2003;3(6):560–568 10.7861/clinmedicine.3-6-560 14994716 PMC4952585

[3] Yoder MC Ingram DA: Endothelial progenitor cell: ongoing controversy for defining these cells and their role in neoangiogenesis in the murine system. *Curr Opin Hematol.* 2009;16(4):269–273 10.1097/MOH.0b013e32832bbcab 19417649

[4] Zeigler BM Sugiyama D Chen M: The allantois and chorion, when isolated before circulation or chorio-allantoic fusion, have hematopoietic potential. *Development.* 2006;133(21):4183–4192 10.1242/dev.02596 17038514

[5] Ueno H Weissman IL: The origin and fate of yolk sac hematopoiesis: application of chimera analyses to developmental studies. *Int J Dev Biol.* 2010;54(6–7):1019–1031 10.1387/ijdb.093039hu 20711980

[6] Gadue P Weiss MJ: Stem cells unscramble yolk sac hematopoiesis. *Blood.* 2009;114(8):1455–1456 10.1182/blood-2009-06-226209 19696206

[7] Klimchenko O Mori M Distefano A: A common bipotent progenitor generates the erythroid and megakaryocyte lineages in embryonic stem cell-derived primitive hematopoiesis. *Blood.* 2009;114(8):1506–1517 10.1182/blood-2008-09-178863 19478046

[8] Geissmann F Manz MG Jung S: Development of monocytes, macrophages, and dendritic cells. *Science.* 2010;327(5966):656–661 10.1126/science.1178331 20133564 PMC2887389

[9] Hanahan D Weinberg RA: Hallmarks of cancer: the next generation. *Cell.* 2011;144(5):646–674 10.1016/j.cell.2011.02.013 21376230

[10] Folkman J: Anti-angiogenesis: new concept for therapy of solid tumors. *Ann Surg.* 1972;175(3):409–416 10.1097/00000658-197203000-00014 5077799 PMC1355186

[11] Asahara T Murohara T Sullivan A: Isolation of putative progenitor endothelial cells for angiogenesis. *Science.* 1997;275(5302):964–967 10.1126/science.275.5302.964 9020076

[12] Purhonen S Palm J Rossi D: Bone marrow-derived circulating endothelial precursors do not contribute to vascular endothelium and are not needed for tumor growth. *Proc Natl Acad Sci U S A.* 2008;105(18):6620–6625 10.1073/pnas.0710516105 18443294 PMC2365563

[13] McGrath K Palis J: Ontogeny of erythropoiesis in the mammalian embryo. *Curr Top Dev Biol.* 2008;82:1–22 10.1016/S0070-2153(07)00001-4 18282515

[14] Sangiorgi F Woods CM Lazarides E: Vimentin downregulation is an inherent feature of murine erythropoiesis and occurs independently of lineage. *Development.* 1990;110(1):85–96 1706980 10.1242/dev.110.1.85

[15] Palis J Robertson S Kennedy M: Development of erythroid and myeloid progenitors in the yolk sac and embryo proper of the mouse. *Development.* 1999;126(22):5073–5084 10529424 10.1242/dev.126.22.5073

[16] Palis J Malik J McGrath KE: Primitive erythropoiesis in the mammalian embryo. *Int J Dev Biol.* 2010;54(6–7):1011–1018 10.1387/ijdb.093056jp 20711979

[17] Dzierzak E Speck NA: Of lineage and legacy: the development of mammalian hematopoietic stem cells. *Nat Immunol.* 2008;9(2):129–136 10.1038/ni1560 18204427 PMC2696344

[18] Yoder MC Hiatt K Dutt P: Characterization of definitive lymphohematopoietic stem cells in the day 9 murine yolk sac. *Immunity.* 1997;7(3):335–344 10.1016/S1074-7613(00)80355-6 9324354

[19] Schlitt HJ Schafers S Deiwick A: Extramedullary erythropoiesis in human liver grafts. *Hepatology.* 1995;21(3):689–696 10.1016/0270-9139(95)90519-7 7533123

[20] Costa-Val R Nunes TA Silva RC: Inhibition of rats extramedullary liver erytropoiesis by hyperbaric oxygen therapy. *Acta Cir Bras.* 2007;22(2):137–141 10.1590/S0102-86502007000200011 17375221

[21] Han J Koh YJ Moon HR: Adipose tissue is an extramedullary reservoir for functional hematopoietic stem and progenitor cells. *Blood.* 2010;115(5):957–964 10.1182/blood-2009-05-219923 19897586

[22] Maliszewski M Majchrzak H Ladzinski P: [Extramedullary erythropoiesis in cerebellar hemangioblastomas]. *Neurol Neurochir Pol.* 1995;29(5):713–722 8584097

[23] Mathews MS Duma CM Brant-Zawadzki M: Extramedullary hematopoeisis within a convexity meningioma. *Surg Neurol.* 2008;69(5):522–525 10.1016/j.surneu.2007.02.017 17714768

[24] Kuhn E Dorji T Rodriguez J: Extramedullary erythropoiesis in chronic subdural hematoma simulating metastatic small round cell tumor. *Int J Surg Pathol.* 2007;15(3):288–291 10.1177/1066896907302534 17652539

[25] Skutelsky E Danon D: An electron microscopic study of nuclear elimination from the late erythroblast. *J Cell Biol.* 1967;33(3):625–635 10.1083/jcb.33.3.625 6036525 PMC2107197

[26] Simpson CF Kling JM: The mechanism of denucleation in circulating erythroblasts. *J Cell Biol.* 1967;35(1):237–245 10.1083/jcb.35.1.237 6061718 PMC2107122

[27] Rajapakse I Perlman MD Scalzo D: The emergence of lineage-specific chromosomal topologies from coordinate gene regulation. *Proc Natl Acad Sci U S A.* 2009;106(16):6679–6684 10.1073/pnas.0900986106 19276122 PMC2654024

[28] Gold P Freedman SO: Specific carcinoembryonic antigens of the human digestive system. *J Exp Med.* 1965;122(3):467–481 10.1084/jem.122.3.467 4953873 PMC2138078

[29] Weinhouse S: Glycolysis, respiration, and anomalous gene expression in experimental hepatomas: G.H.A. Clowes memorial lecture. *Cancer Res.* 1972;32(10):2007–2016 4343003

[30] Abelev GI: Alpha-fetoprotein as a marker of embryo-specific differentiations in normal and tumor tissues. *Transplant Rev.* 1974;20:3–37 4135841 10.1111/j.1600-065x.1974.tb00139.x

[31] Hammarström S: The carcinoembryonic antige, and (CEA) family: structures, suggested functions and expression in normal and malignant tissues. *Semin Cancer Biol.* 1999;9(2):67–81 10.1006/scbi.1998.0119 10202129

[32] Sood AK Fletcher MS Hendrix MJ: The embryonic-like properties of aggressive human tumor cells. *J Soc Gynecol Investig.* 2002;9(1):2–9 11839501 10.1016/s1071-5576(01)00147-2

[33] McDonald DM Munn L Jain RK: Vasculogenic mimicry: how convincing, how novel, and how significant? *Am J Pathol.* 2000;156(2):383–388 10.1016/S0002-9440(10)64740-2 10666365 PMC1850027

[34] Li L Connelly MC Wetmore C: Mouse embryos cloned from brain tumors. *Cancer Res.* 2003;63(11):2733–2736 12782575

[35] Pierce GB Aguilar D Hood G: Trophectoderm in control of murine embryonal carcinoma. *Cancer Res.* 1984;44(9):3987–3996 6744314

[36] Potten CS Loeffler M: Stem cells: attributes, cycles, spirals, pitfalls and uncertainties. Lessons for and from the crypt. *Development.* 1990;110(4):1001–1020 2100251 10.1242/dev.110.4.1001

[37] Loeffler M Roeder I: Tissue stem cells: definition, plasticity, heterogeneity, self-organization and models–a conceptual approach. *Cells Tissues Organs.* 2002;171(1):8–26 10.1159/000057688 12021488

[38] Roeder I Loeffler M: A novel dynamic model of hematopoietic stem cell organization based on the concept of within-tissue plasticity. *Exp Hematol.* 2002;30(8):853–861 12160836 10.1016/s0301-472x(02)00832-9

[39] Loeffler M Roeder I: Conceptual models to understand tissue stem cell organization. *Curr Opin Hematol.* 2004;11(2):81–87 15257023 10.1097/01.moh.0000133648.83991.af

[40] Lander AD: The 'stem cell' concept: is it holding us back? *J Biol.* 2009;8(8):70 10.1186/jbiol177 19769787 PMC2776917

[41] Salipante SJ Kas A McMonagle E: Phylogenetic analysis of developmental and postnatal mouse cell lineages. *Evol Dev.* 2010;12(1):84–94 10.1111/j.1525-142X.2009.00393.x 20156285 PMC2824914

[42] Glauche I Moore K Thielecke L: Stem cell proliferation and quiescence–two sides of the same coin. *PLoS Comput Biol.* 2009;5(7):e1000447 10.1371/journal.pcbi.1000447 19629161 PMC2704962

[43] Witkiewicz H Oh P Schnitzer JE: II. Capsular vaso-mimicry formed by transgenic mammary tumor spheroids implanted ectopically into mouse dorsal skin fold - cellular mechanisms of metastasis [v1; ref status: awaiting peer review, http://f1000r.es/9z]. *F1000 Res.* 2013;2(9). 10.12688/f1000research.2-9.v2 PMC3869488 24555024

[44] Torres Filho IP Hartley-Asp B Borgström P: Quantitative angiogenesis in a syngeneic tumor spheroid model. *Microvasc Res.* 1995;49(2):212–226 10.1006/mvre.1995.1017 7541506

[45] Frost GI Borgström P: Real time *in vivo* quantitation of tumor angiogenesis. *Methods Mol Med.* 2003;85:65–78 10.1385/1-59259-380-1:65 12710198

[46] Som PM Brandwein M Silvers AR: Sialoblastoma (embryoma): MR findings of a rare pediatric salivary gland tumor. *AJNR Am J Neuroradiol.* 1997;18(5):847–850 9159361 PMC8338107

[47] Liao MJ Zhang CC Zhou B: Enrichment of a population of mammary gland cells that form mammospheres and have *in vivo* repopulating activity. *Cancer Res.* 2007;67(17):8131–8138 10.1158/0008-5472.CAN-06-4493 17804725

[48] Nanni P Pupa SM Nicoletti G: p185(neu) protein is required for tumor and anchorage-independent growth, not for cell proliferation of transgenic mammary carcinoma. *Int J Cancer.* 2000;87(2):186–194 10.1002/1097-0215(20000715)87:2<186::AID-IJC5>3.0.CO;2-1 10861472

[49] Cuadros C Dominguez AL Frost GI: Cooperative effect between immunotherapy and antiangiogenic therapy leads to effective tumor rejection in tolerant Her-2/neu mice. *Cancer Res.* 2003;63(18):5895–5901 14522915

[50] Oh P Borgström P Witkiewicz H: Live dynamic imaging of caveolae pumping targeted antibody rapidly and specifically across endothelium in the lung. *Nat Biotechnol.* 2007;25(3):327–337 10.1038/nbt1292 17334358 PMC1979160

[51] Hanaichi T Sato T Iwamoto T: A stable lead by modification of Sato's method. *J Electron Microsc (Tokyo).* 1986;35(3):304–306 2440973

[52] Tokuyasu KT: Application of cryoultramicrotomy to immunocytochemistry. *J Microsc.* 1986;143(Pt 2):139–149 10.1111/j.1365-2818.1986.tb02772.x 3531524

[53] Tober J Koniski A McGrath KE: The megakaryocyte lineage originates from hemangioblast precursors and is an integral component both of primitive and of definitive hematopoiesis. *Blood.* 2007;109(4):1433–1441 10.1182/blood-2006-06-031898 17062726 PMC1794060

[54] Rieger MA Smejkal BM Schroeder T: Improved prospective identification of megakaryocyte-erythrocyte progenitor cells. *Br J Haematol.* 2009;144(3):448–451 10.1111/j.1365-2141.2008.07419.x 19036095

[55] Kim M Cooper DD Hayes SF: Rhodamine-123 staining in hematopoietic stem cells of young mice indicates mitochondrial activation rather than dye efflux. *Blood.* 1998;91(11):4106–4117 9596656

[56] Parise LV Smyth SS Coller BS: Platelet morphology biochemistry and function. Chapter 111 in Williams Hematology.2001;1357–1408(ISBN 0-07-116293-3). Reference Source

[57] Ford Bainton D: Morphology of neutrophils, eosinophils, and basophils. Chapter 64 in Williams Hematology.2001;729–743(ISBN 0-07-116293-3). Reference Source

[58] Subasinghe W Spence DM: Simultaneous determination of cell aging and ATP release from erythrocytes and its implications in type 2 diabetes. *Anal Chim Acta.* 2008;618(2):227–233 10.1016/j.aca.2008.04.061 18513544 PMC2779697

[59] Dvorak AM Kohn S Morgan ES: The vesiculo-vacuolar organelle (VVO): a distinct endothelial cell structure that provides a transcellular pathway for macromolecular extravasation. *J Leukoc Biol.* 1996;59(1):100–115 8558058

[60] Idé C Saito T: Adenosine triphosphatase activity of cutaneous nerve fibers. *Histochemistry.* 1980;65(2):83–92 10.1007/BF00493157 6102083

[61] Nasu F Inomata K: Ultracytochemical demonstration of Ca2(+)-ATPase activity in the rat saphenous artery and its innervated nerve terminal. *J Electron Microsc (Tokyo).* 1990;39(6):487–491 2151280

[62] Fujimoto T: Calcium pump of the plasma membrane is localized in caveolae. *J Cell Biol.* 1993;120(5):1147–1157 10.1083/jcb.120.5.1147 8382206 PMC2119723

[63] Schnitzer JE Oh P Jacobson BS: Caveolae from luminal plasmalemma of rat lung endothelium: microdomains enriched in caveolin, Ca(2+)-ATPase, and inositol trisphosphate receptor. *Proc Natl Acad Sci U S A.* 1995;92(5):1759–1763 10.1073/pnas.92.5.1759 7878055 PMC42599

[64] Brown JM Giaccia AJ: The unique physiology of solid tumors: opportunities (and problems) for cancer therapy. *Cancer Res.* 1998;58(7):1408–1416 9537241

[65] Bertossi M Roncali L Nico B: Ultrastructure and permeability of immature neural and extraneural blood vessels. *Biol Struct Morphog.* 1990;3(1):37–44 2091805

[66] Wolff JR Goerz C Bär T: Common morphogenetic aspects of various organotypic microvascular patterns. *Microvasc Res.* 1975;10(3):373–395 10.1016/0026-2862(75)90040-0 1214601

[67] Gurdon JB: The developmental capacity of nuclei taken from intestinal epithelium cells of feeding tadpoles. *J Embryol Exp Morphol.* 1962;10:622–640 13951335

[68] Takahashi K Yamanaka S: Induction of pluripotent stem cells from mouse embryonic and adult fibroblast cultures by defined factors. *Cell.* 2006;126(4):663–676 10.1016/j.cell.2006.07.024 16904174

[69] Krawczyk A Izdebska M Grzanka A: [The influence of hyperglycemia on functions of endothelial progenitor cells]. *Pol Merkur Lekarski.* 2009;26(153):245–247 19388542

[70] Blau HM Baltimore D: Differentiation requires continuous regulation. *J Cell Biol.* 1991;112(5):781–783 10.1083/jcb.112.5.781 1999456 PMC2288865

[71] Tsang AP Fujiwara Y Hom DB: Failure of megakaryopoiesis and arrested erythropoiesis in mice lacking the GATA-1 transcriptional cofactor FOG. *Genes Dev.* 1998;12(8):1176–1188 9553047 10.1101/gad.12.8.1176 PMC316724

[72] Randrianarison-Huetz V Laurent B Bardet V: Gfi-1B controls human erythroid and megakaryocytic differentiation by regulating TGF-beta signaling at the bipotent erythro-megakaryocytic progenitor stage. *Blood.* 2010;115(14):2784–2795 10.1182/blood-2009-09-241752 20124515

[73] Ribatti D: Hemangioblast does exist. *Leuk Res.* 2008;32(6):850–854 10.1016/j.leukres.2007.12.001 18192009

[74] Soda Y Marumoto T Friedmann-Morvinski D: Transdifferentiation of glioblastoma cells into vascular endothelial cells. *Proc Natl Acad Sci U S A.* 2011;108(11):4274–4280 10.1073/pnas.1016030108 21262804 PMC3060261

[75] Nishikawa SI Nishikawa S Kawamoto H: *In vitro* generation of lymphohematopoietic cells from endothelial cells purified from murine embryos. *Immunity.* 1998;8(6):761–769 10.1016/S1074-7613(00)80581-6 9655490

[76] Robin C Durand C: The roles of BMP and IL-3 signaling pathways in the control of hematopoietic stem cells in the mouse embryo. *Int J Dev Biol.* 2010;54(6–7):1189–1200 10.1387/ijdb.093040cr 20711995

[77] Kitchen SM Kempston T Alberts AS: Endometrial Hyperplasia Resulting from Myeloid-Specific Targeting of the Tumor Suppressor APC: Epithelium Arising from Bone Marrow Progenitors? 2009;5–5

[78] Zovein AC Hofmann JJ Lynch M: Fate tracing reveals the endothelial origin of hematopoietic stem cells. *Cell Stem Cell.* 2008;3(6):625–636 10.1016/j.stem.2008.09.018 19041779 PMC2631552

[79] Lach B Gregor A Rippstein P: Angiogenic histogenesis of stromal cells in hemangioblastoma: ultrastructural and immunohistochemical study. *Ultrastruct Pathol.* 1999;23(5):299–310 10.1080/019131299281446 10582267

[80] Bjornson CR Rietze RL Reynolds BA: Turning brain into blood: a hematopoietic fate adopted by adult neural stem cells *in vivo*. *Science.* 1999;283(5401):534–537 10.1126/science.283.5401.534 9915700

[81] Jackson KA Mi T Goodell MA: Hematopoietic potential of stem cells isolated from murine skeletal muscle. *Proc Natl Acad Sci U S A.* 1999;96(25):14482–14486 10.1073/pnas.96.25.14482 10588731 PMC24462

[82] Gussoni E Soneoka Y Strickland CD: Dystrophin expression in the mdx mouse restored by stem cell transplantation. *Nature.* 1999;401(6751):390–394 10.1038/43919 10517639

[83] Badorff C Brandes RP Popp R: Transdifferentiation of blood-derived human adult endothelial progenitor cells into functionally active cardiomyocytes. *Circulation.* 2003;107(7):1024–1032 10.1161/01.CIR.0000051460.85800.BB 12600917

[84] Minana MD Carbonell-Uberos F Mirabet V: IFATS collection: Identification of hemangioblasts in the adult human adipose tissue. *Stem Cells.* 2008;26(10):2696–2704 10.1634/stemcells.2007-0988 18450825

[85] Wurmser AE Nakashima K Summers RG: Cell fusion-independent differentiation of neural stem cells to the endothelial lineage. *Nature.* 2004;430(6997):350–356 10.1038/nature02604 15254537

[86] Ogawa M Larue AC Watson PM: Hematopoietic stem cell origin of connective tissues. *Exp Hematol.* 2010;38(7):540–547 10.1016/j.exphem.2010.04.005 20412832

[87] Witkiewicz H Oh P Schnitzer JE: III. Cellular ultrastructures *in situ* as key to understanding tumor energy metabolism - biological significance of the Warburg effect [v1; ref status: awaiting peer review, http://f1000r.es/a0]. *F1000 Res.* 2013;2(10). 10.12688/f1000research.2-10.v1 PMC3829121 24358890

[88] Namazi MR Fallahzadeh MK Schwartz RA: Strategies for prevention of scars: what can we learn from fetal skin? *Int J Dermatol.* 2011;50(1):85–93 10.1111/j.1365-4632.2010.04678.x 21039435

[89] Wu DC Paulson RF: Hypoxia regulates BMP4 expression in the murine spleen during the recovery from acute anemia. *PLoS One.* 2010;5(6):e11303 10.1371/journal.pone.0011303 20585586 PMC2892039

[90] Goldman O Feraud O Boyer-Di Ponio J: A boost of BMP4 accelerates the commitment of human embryonic stem cells to the endothelial lineage. *Stem Cells.* 2009;27(8):1750–1759 10.1002/stem.100 19544443

[91] Pearson S Sroczynska P Lacaud G: The stepwise specification of embryonic stem cells to hematopoietic fate is driven by sequential exposure to Bmp4, activin A, bFGF and VEGF. *Development.* 2008;135(8):1525–1535 10.1242/dev.011767 18339678

[92] Nardelli J Thiesson D Fujiwara Y: Expression and genetic interaction of transcription factors GATA-2 and GATA-3 during development of the mouse central nervous system. *Dev Biol.* 1999;210(2):305–321 10.1006/dbio.1999.9278 10357893

[93] Olive PL Vikse C Trotter MJ: Measurement of oxygen diffusion distance in tumor cubes using a fluorescent hypoxia probe. *Int J Radiat Oncol Biol Phys.* 1992;22(3):397–402 10.1016/0360-3016(92)90840-E 1735668

[94] Si ZC Liu J: What "helps" tumors evade vascular targeting treatment? *Chin Med J (Engl).* 2008;121(9):844–849 18701052

[95] Eichmann A Makinen T Alitalo K: Neural guidance molecules regulate vascular remodeling and vessel navigation. *Genes Dev.* 2005;19(9):1013–1021 10.1101/gad.1305405 15879551

[96] El Hallani S Boisselier B Peglion F: A new alternative mechanism in glioblastoma vascularization: tubular vasculogenic mimicry. *Brain.* 2010;133(Pt 4):973–982 10.1093/brain/awq044 20375132 PMC4861203

[97] Palmer TD Willhoite AR Gage FH: Vascular niche for adult hippocampal neurogenesis. *J Comp Neurol.* 2000;425(4):479–494 10.1002/1096-9861(20001002)425:4<479::AID-CNE2>3.0.CO;2-3 10975875

[98] Mirzadeh Z Merkle FT Soriano-Navarro M: Neural stem cells confer unique pinwheel architecture to the ventricular surface in neurogenic regions of the adult brain. *Cell Stem Cell.* 2008;3(3):265–278 10.1016/j.stem.2008.07.004 18786414 PMC2613692

[99] Tavazoie M van der V Silva-Vargas V: A specialized vascular niche for adult neural stem cells. *Cell Stem Cell.* 2008;3(3):279–288 10.1016/j.stem.2008.07.025 18786415 PMC6864413

[100] Shen Q Wang Y Kokovay E: Adult SVZ stem cells lie in a vascular niche: a quantitative analysis of niche cell-cell interactions. *Cell Stem Cell.* 2008;3(3):289–300 10.1016/j.stem.2008.07.026 18786416 PMC2747473

[101] Yang XT Bi YY Feng DF: From the vascular microenvironment to neurogenesis. *Brain Res Bull.* 2011;84(1):1–7 10.1016/j.brainresbull.2010.09.008 20850508

[102] Alby L Auerbach R: Differential adhesion of tumor cells to capillary endothelial cells *in vitro*. *Proc Natl Acad Sci U S A.* 1984;81(18):5739–5743 10.1073/pnas.81.18.5739 6592584 PMC391786

[103] Auerbach R Alby L Morrissey LW: Expression of organ-specific antigens on capillary endothelial cells. *Microvasc Res.* 1985;29(3):401–411 10.1016/0026-2862(85)90028-7 2582227

[104] Auerbach R Lu WC Pardon E: Specificity of adhesion between murine tumor cells and capillary endothelium: an *in vitro* correlate of preferential metastasis *in vivo*. *Cancer Res.* 1987;47(6):1492–1496 3815350

[105] Pasqualini R Ruoslahti E: Organ targeting *in vivo* using phage display peptide libraries. *Nature.* 1996;380(6572):364–366 10.1038/380364a0 8598934

[106] St Croix B Rago C Velculescu V: Genes expressed in human tumor endothelium. *Science.* 2000;289(5482):1197–1202 10.1126/science.289.5482.1197 10947988

[107] Ruoslahti E Rajotte D: An address system in the vasculature of normal tissues and tumors. *Annu Rev Immunol.* 2000;18:813–827 10.1146/annurev.immunol.18.1.813 10837076

[108] Reese TS Karnovsky MJ: Fine structural localization of a blood-brain barrier to exogenous peroxidase. *J Cell Biol.* 1967;34(1):207–217 10.1083/jcb.34.1.207 6033532 PMC2107213

