# Supplementary material for: I. Embryonal vasculature formation recapitulated in transgenic mammary tumor spheroids implanted pseudo-orthotopicly into mouse dorsal skin fold: the organoblasts concept
Source: F1000Res. 2013 Jul 11;2:8. Originally published 2013 Jan 10. [Version 2] doi: 10.12688/f1000research.2-8.v2 (PMC3938277; doi:10.12688/f1000research.2-8.v2)
Supplement: Peer review history

# Peer review: Referee response for version 2

**Published:** 2014-01-28

**Authors:** Karolina Kucharova

**Affiliations:** 1 Sanford-Burnham Medical Research Institute, La Jolla, CA, USA

I have read this submission. I believe that I have an appropriate level of expertise to confirm that it is of an acceptable scientific standard.

# Peer review: Referee response for version 2

**Published:** 2013-07-22

**Authors:** Anita Bandrowski

**Affiliations:** 1 Division of Biological Sciences, University of California, San Diego, La Jolla, CA, USA

The authors addressed the main point of my criticism adequately. The number of animals in the report is now more clearly defined in table 1 and they are adequate to support the conclusions. 

The other minor points seem to have been addressed throughout the manuscript.

I have read this submission. I believe that I have an appropriate level of expertise to confirm that it is of an acceptable scientific standard.

# Peer review: Referee response for version 2

**Published:** 2013-07-15

**Authors:** Louise Purton

**Affiliations:** 1 Stem Cell Regulation Unit, St Vincent’s Institute, Fitzroy, VIC, Australia

The authors have not sufficiently addressed my concerns in their response to my original assessment. My concerns remain the same.

I have read this submission. I believe that I have an appropriate level of expertise to state that I do not consider it to be of an acceptable scientific standard, for reasons outlined above.

# Peer review: Referee response for version 1

**Published:** 2013-05-30

**Authors:** Karolina Kucharova

**Affiliations:** 1 Sanford-Burnham Medical Research Institute, La Jolla, CA, USA

The title: “I. Embryonal vasculature formation recapitulated in transgenic mammary tumor spheroids implanted pseudo-orthotopicly into mouse dorsal skin fold: the organoblasts concept” is appropriate for the content of the article.  

The abstract represents a suitable summary of the work.

The methods are well documented, except there is little information about the chamber that was implanted – that portion needs to be expanded (e.g. explain the chamber design, dimensions, material, the pore sizes). How the tumor spheroids were implanted into the chamber (e.g. injected by needle). Using the results section, I only assume that the chamber was made from glass and covered by a hyaline membrane. 

The article is well constructed and is bringing a different opinion about angiogenesis in a tumor microenvironment. I appreciate that the authors moved their observation from *in vitro* to *in vivo* studies and emphasize the fact that the processes in the tumor microenvironment are very dynamic.

I appreciate that the authors studied cell morphology during their analyses rather than relying solely on immuno-markers.

If the authors prefer to use the original black and white microphotographs, then I suggest adding arrows, arrowheads and other symbols to the figures, figure legends, and text to indicate exactly which cells are being discussed – e.g. megakaryocytes or erythroblast in figure 1A.  This type of correction should be carried on to all other figures.  

Or the authors may switch the B/W Figure 1 with a color Figure S1 (there are the different cell types indicated by the colors). 

In figure 5 and S5, I suggest adding arrows pointing at GFP positive structures. 

In figure 10A and S10A, change the scale unit ‘µM’ to ‘µm’.

The discussion seems longer than needed – I suggest that it should be cut to focus on the current results.

I have read this submission. I believe that I have an appropriate level of expertise to confirm that it is of an acceptable scientific standard.

# Response

**Published:** 2013-07-03

**Authors:** Halina Witkiewicz

**Affiliations:** PRISM, USA

The scale bar unit in Figures 10 [A] & S10 [A] has been corrected. The details of setting up the chambers have been described in references 44, 45 & 50.

# Peer review: Referee response for version 1

**Published:** 2013-05-08

**Authors:** Anita Bandrowski

**Affiliations:** 1 Division of Biological Sciences, University of California, San Diego, La Jolla, CA, USA

The paper entitled 'I. Embryonal vasculature formation recapitulated in transgenic mammary tumour spheroids implanted pseudo-orthotopicly into mouse dorsal skin fold: the organoblasts concept' by Witkiewicz, Oh and Schnitzer presents a novel and interesting view of vasculature development in solid tumours. The notion that tumour cells become organized into a pseudo organ, complete with its' own division of labour is compelling. The methods and results reported herein show a significant meticulous scholarship in this group. The work is of a high quality and the paper is generally well written.

The reporting of methods is refreshingly complete, but a few things should be clarified. In a recent announcement of Nature's policies on methods reporting (see http://www.nature.com/authors/policies/checklist.pdf) several items are pointed out as critical, which the authors address; they provide stock numbers for animals, catalogue numbers for significant reagents, the order of procedures and most of the other characteristics specified as significant. The parts of the methods that must be clarified are: The n's could be made explicit throughout the paper; and the reasons that incubation in addition to graft tissue differed between figures 1-8 vs 9+ should be clarified by the authors before final approval.

As the first reviewer correctly points out, the interpretation of ultra-structural data can be an art, and it is my opinion that the only way to get a good sense of these figures is to bring the community in, allowing them to see the full size images and manipulate them. There are projects that collect images that are full size and allow the community to view and manipulate the data sets, comparing images from different data sets and deploying automated tools to align them (the cell: an image library cellimagelibrary.org or the Cell Centered DataBase ccdb.ucsd.edu). While this step is not required, it may be a good way to increase the visibility of the data and allow multiple parties to weigh in on interpretation.

The conclusions presented in the study are coherent with the data, but at odds with a prevailing theory. The authors bring up the point that "the views on tumour neo-angiogenesis have remained polarized and we have yet to stop tumour growth" in no uncertain terms bringing ire from the community. The statement may not be completely fair, but given the lack of ability of independent researchers to reproduce most significant cancer studies ( Nature 483, 531–533) it may not be unfounded and again it may generate controversy, spurring others to repeat the work presented here. If the work stands the test of time and the ire of the community, the implications will be profound.

A set of minor points are found below.

RT is not defined as acronym, seems to be room temperature, but should be stated

Perhaps a table of acronyms found in the paper would help in readability?

Please rewrite this sentence to clarify your point;

'The presence of a composite dense body, characteristic of platelets ^56^, in an extra-cellular matrix (ECM) producer indicated that the ECM building cell was related to megakaryocyte (Figure 1[E & F]).'

I have read this submission. I believe that I have an appropriate level of expertise to confirm that it is of an acceptable scientific standard, however I have significant reservations, as outlined above.

# Response

**Published:** 2013-07-03

**Authors:** Halina Witkiewicz

**Affiliations:** PRISM, USA

This and the two accompanying articles report results of basic research on tumor biology. The rules regarding ‘n’s and controls required for pre-clinical pharmacological studies do not apply here because we did not test any treatment or hypothesis for a mechanism of action of a potential new drug. The article describes phenomena that *in vivo* occur simultaneously, side by side, because unlike in embryo, the tumors keep growing but never mature. Total 5 mice were used to make observations that were unprecedented and consistently corrected the erroneous belief lasting since 1967 regarding the mechanism of erythrocyte enucleation by nuclear expulsion ( Skutelsky & Danon, *J Cell Biol* (1967); Simpson & Kling, *J Cell Biol* (1967) ). In those two publications, the numbers of mice or dogs used for the ultrastructural analysis *in situ* were not mentioned. For our purposes, the numbers of animals with pseudo-orthotopicly and ectopicly implanted tumor spheroids (n=3 and n=2, respectively) appear adequate. A link to the image library will be provided when the images are deposited. Other critical comments made by the referee have been addressed in the amended version of the article.

# Peer review: Referee response for version 1

**Published:** 2013-01-14

**Authors:** Louise Purton

**Affiliations:** 1 Stem Cell Regulation Unit, St Vincent’s Institute, Fitzroy, VIC, Australia

The authors rely heavily on their own identification of ultrastructure components of cells without any confirmation of the identity of the cells by immunostaining. Furthermore, all of the immunostaining they do show is questionable and likely non-specific staining (in the materials and methods the appropriate species-matched isotype control is not listed, the one that is listed is of incorrect species). While megakaryocytes and erythrocytes are easily morphologically identifiable, I disagree with their interpretation of the data at 5 days, when they state that no endothelial cells are visible. My opinion is that an EC is visible in Figure 1C at the bottom (they claim this is a megakaryocyte). The authors are strongly advised to read papers by Leon Weiss, who did excellent TEM studies of mouse bone marrow and is the expert in this field. Without confirmation of the different cell types in this study with specific immunostaining their conclusions are not valid. Larger numbers of mice at each time point are also required to show reproducibility of findings.

I have read this submission. I believe that I have an appropriate level of expertise to state that I do not consider it to be of an acceptable scientific standard, for reasons outlined above.

# Response

**Published:** 2013-04-10

**Authors:** Halina Witkiewicz

**Affiliations:** PRISM, USA

**Identifying cells** 

The referee’s notion that we rely heavily on cellular morphology (at the ultrastructural level) is accurate. To that experimental approach we devoted the opening section of the Discussion in the accompanying article ( III) because we believe it is the strength of our study. 

The referee’s generic skepticism with regard to usefulness of morphological features for identifying cell phenotypes is inconsistent with her other statements. On one hand she states that “Without confirmation of the different cell types … with specific immunostaining… conclusions are not valid” and on the other that “… megakaryocytes and erythrocytes are easily morphologically identifiable…” and goes on to identify a cell in Figure 2 as endothelial (EC) - without confirmation with the specific immunostaining. However, the cell in question does not fulfill the criteria that we used for the EC phenotype, namely: a flattened shape, polarized cell membrane with abundant caveolae (mostly on luminal surface) and attachment to collagen or basement membrane (on abluminal surface), collagen synthesis and occasionally seen Weibel-Palade bodies (page 10 of the accompanying article III). 

Moreover, *in vivo *mature -thelial cells of any kind (endo-, epi- or meso-) have tight contacts with one another. That is how ECs can be identified also in absence of erythrosomes (in sections of tissues fixed by perfusion). In contrast, the hallmarks of megakaryocyte are: large nuclei often containing double nucleoli, large asymmetrically distributed irregularly shaped cytoplasm and satellite platelets (Figure 1 [A] and 2 [B]). The referee does not specify criteria by which the cell in Figure 2 was in her opinion an EC and not a megakaryocyte. 

**Antibodies** 

The referee questions specificity of the antibodies that we used: GFP-specific rabbit polyclonal IgG (ab290) from Abcam; calmyrin-specific rabbit polyclonal IgG (11823-1-AP) from ProteinTech Group, Inc. and CD34-specific monoclonal IgG _2a_ (sc-18917) from Santa Cruz. All those vendors validated the specificity of the antibodies in ways presented in their datasheets. Abcam and Santa Cruz also provided references to published work by authors who used the antibodies successfully.

Nevertheless, each experimental setting requires controls addressing issues specific for that setting. Out of 150 publications that used the GFP-specific antibody only one had the species matched isotype control and out of 15 that used the CD34-specific antibody also only one had it. Both reported flow cytometry studies. 

For *in situ* analysis the best controls are the internal ones, demonstrating variable pattern of staining that correlates with particular ultrastructures as expected based on the experimental design. We showed those. The pattern of GFP-specific immunostaining depended on the promoter controlling expression of the inserted GFP coding sequence. In case of the ubiquitin promoter the label was ubiquitous, including erythrosomes, whereas in case of the histone H2B promoter the label was associated with chromatin, regardless of the cell cycle phase (inter-phase nucleus or mitotic chromosomes), although not in all cells because neither the host nor the graft donor mouse contained the GFP. Therefore the erythrosome labeled with anti-ubiquitin antibody and internalized by unlabeled macrophage must have been of the graft origin (Figure 15 [A, B]). Similarly, the unlabeled fibroblast next to the labeled dividing tumor cell must have been of the host origin (Figure 2 [F] in the accompanying article II). Thus the same antibody produced different staining patterns in sections from differently handled host mice. Both were expected and easy to explain. 

The variable distribution of calmyrin-associated Au grains correlated with the level of morphological complexity in the evolving phenotypes of HSC lineage (Figure 6 EFI & Figure 8). In emerging erythrosomes they were randomly dispersed initially and in more mature forms they appeared in distinct clusters, the calmyrites, whereas the CD34 label vanished from the nuclear area at the start of the nucleo-cytoplasmic conversion (Figure 4 [F]) and was not found in erythrosomes. 

None of the three antibodies were essential in identifying particular phenotypes. CD34, thought of as a primitive stem cell marker, was present in various differentiated cells of HSC lineage as well, and the distinct clusters of Au particles attributed to calmyrites were present in even greater variety of differentiated cell types, including nonmalignant rat epithelium. Such clusters were not seen extracellularly. Finally, both the anti-GFP and anti-calmyrin antibodies were rabbit polyclonal IgGs and therefore, represented the species matched isotype controls for each other. 

**Numbers** Our goal was accomplished by generating unprecedented photographic documentation of spontaneous inter-cellular relations leading to the vasculature formation. The animals were not subjected to any kind of treatment except implantation of the tumor. The nature of the study was exploratory and observational therefore the results were qualitative. The images represented raw data, i.e. a direct demonstration of the phenomena that had actually occurred *in vivo*. The conventional thinking in terms of comparing treated and control groups over a period of time to measure an anticipated effect and to evaluate its statistical significance was not applicable here. The time progression was not critical either, in contrast to the embryonal growth and development, because the tumor never matures. 

In our model the cells were responding to locally variable microenvironment therefore they were not functionally synchronized. Consequently a range of different stages of vasculature morphogenesis could be found simultaneously within single sections. To extend that range we did have two different times of incubating tumors in the skin fold chambers: one and three weeks. Indeed, different yet overlapping sets of options were found. However, in tumors grown for three weeks without the graft we found the earliest stages of vasculature morphogenesis (Figure 1) repeated in necrotic areas that were being repopulated. Thus the range of options was well covered. 

To illustrate our conclusions we selected representative images after careful analysis of many (several hundreds per mouse at various magnifications). We stopped the analysis when the findings became redundant and we understood the observed phenomena. In the literature the number of studied animals tends to be inversely proportional to complexity of assays. For example, one study using ab290 involved animal groups of variable size: n = 18 for flow cytometry and n = 6 for histology ( PM:15883207). For electron microscopy based studies it is common to use samples from small number of individuals ( PM:20439620). In all five mice that we used (in this and the accompanying articles) the fundamental cellular mechanism of initiating the vasculature formation turned out to be the same. Thus, the number of animals was adequate for the goal of this particular study. Increasing the number of sacrificed animals above the necessary minimum, just for the number sake, would be superfluous and against the animal welfare rules.
